# Solar-light-driven LaFe*_x_*Ni_1−_*_x_*O_3_ perovskite oxides for photocatalytic Fenton-like reaction to degrade organic pollutants

**DOI:** 10.3762/bjnano.13.79

**Published:** 2022-09-05

**Authors:** Chao-Wei Huang, Shu-Yu Hsu, Jun-Han Lin, Yun Jhou, Wei-Yu Chen, Kun-Yi Andrew Lin, Yu-Tang Lin, Van-Huy Nguyen

**Affiliations:** 1 Department of Engineering Science, National Cheng Kung University, Tainan 70101, Taiwan; 2 Department of Chemical and Materials Engineering, National Kaohsiung University of Science and Technology, Kaohsiung 80778, Taiwan; 3 Department of Materials Engineering, National Pingtung University of Science and Technology, No.1, Xuefu Rd., Neipu Township, Pingtung County 912, Taiwan; 4 i-Center for Advanced Science and Technology (iCAST), Innovation and Development Center of Sustainable Agriculture, Department of Environmental Engineering, National Chung Hsing University, Taichung 402227, Taiwan; 5 Department of Chemical and Biological Engineering, University of Colorado Boulder, Boulder, Colorado 80309, United States; 6 Chettinad Hospital and Research Institute, Chettinad Academy of Research and Education (CARE), Chengalpattu district, Kelambakkam, Tamil Nadu, 603103, India

**Keywords:** LaFeO_3_, LaNiO_3_, methylene blue (MB), perovskite oxides, photocatalyst

## Abstract

LaFe*_x_*Ni_1−_*_x_*O_3_ perovskite oxides were prepared by the sol–gel method under various conditions, including different pH values (pH 0 and pH 7) and different calcination temperatures (500–800 °C) as well as different Fe/Ni ratios (1/9, 3/7, 5/5, 7/3, 9/1). The samples were examined by XRD, DRS, BET, and SEM to reveal their crystallinity, light-absorption ability, specific surface area, and surface features, respectively. The photocatalytic Fenton reaction was conducted using various LaFe*_x_*Ni_1−_*_x_*O_3_ perovskite oxides to decompose the methylene blue molecules. Accordingly, the synthesis condition of pH 0, calcination temperature at 700 °C, and Fe/Ni ratio = 7/3 could form LaFe_0.7_Ni_0.3_O_3_ perovskite oxides as highly efficient photocatalysts. Moreover, various conditions during the photocatalytic degradation were verified, such as pH value, catalyst dosage, and the additional amount of H_2_O_2_. LaFe_0.7_Ni_0.3_O_3_ perovskite oxides could operate efficiently under pH 3.5, catalyst dosage of 50 mg/150 mL, and H_2_O_2_ concentration of 133 ppm to decompose the MB dye in the 1st order kinetic rate constant of 0.0506 s^−1^.

## Introduction

With the advancement of science and technology, the world's population is increasing, leading to the fact that factories are consuming more and more resources. Water inevitably plays a vital role in industrial development among the demanded resources. According to the World Resources Institute (WRI), the demand for freshwater has continued to rise since the 1960s [1]. The inseparable relationship between water and human urban economic activities has been strong. Particularly, agricultural irrigation and animal husbandry have consumed the world's largest water. As the global population increases, the water demand for agricultural planting also increases yearly [2]. Moreover, there is abundant industrial wastewater produced [3]. It originates from the increased demand for electricity, fuels, textiles, and other related industries that consume water [4]. However, it leads to a severe environmental issue due to a large amount of discharged wastewater. For example, domestic wastewater mainly includes organic pollutants from excrement, clothing, cleaning lotion, etc. On the other hand, wastewater discharged from the industry often contains biological drugs, such as antibiotics and pesticides. These drug residues in various industries would enter the drinking water source. It threatens human health and makes bacteria and viruses resistant to drugs, significantly impacting the environment [5]. Notably, wastewater without proper solutions would eventually significantly affect natural ecology and people’s quality of life.

Dyes are widely used in various living areas, such as paint, leather, textiles, oil wax, etc. Accordingly, a large amount of dye wastewater is produced every day. Dye wastewater refers to dyes remaining in the aqueous solution during the textile process. The amount of dye wastewater is enormous and has gradually become the main wastewater discharged in the industry [6]. At present, dyes are mainly divided into azo dyes, thiazine dyes, acridine dyes, and aryl methane dyes. Due to their complex chemical structure and high chromophore, it is not conducive to most biological and physical treatments. Thus, wastewater is regarded as a threat to the environment and health. As environmental awareness is gradually increasing, many countries are currently starting to control the use of harmful azo dyes [7].

On the other hand, pharmaceuticals' personal care products (PPCPs) are also sources of organic pollutants in wastewaters. PPCPs cover prescription drugs, nutritious foods, and personal health products that may cause environmental impacts. Among the medicines, tetracycline is antibiotics, which belong to a sub-category of natural or semi-synthetic polyketides. Tetracycline consists of a linear tetracycline nucleus, exhibiting antibacterial activity, which may affect the biological system after discharging [8]. As the population has become dense, vigorous industrial activities, and the animal husbandry industry is relatively developed nowadays, leading to the wide use of antibiotics and drugs. However, most of these substances are eventually released into rivers or oceans, considerably impacting the domestic water and aquatic environments [9].

Several methods deal with wastewater with organic pollutants, such as adsorption, coagulation, filtration, and chemical and biochemical oxidation [10–11]. Advanced oxidation processes (AOPs) have recently attracted attention due to their simple operation, low cost, and potentially high effectiveness. AOPs are the technologies that use various chemical methods to treat wastewater to purify water quality, such as electrochemical oxidation [12], Fenton method [13], ozonation [14], and photocatalysis [15]. They can achieve a fast reaction rate and extremely high organic removal ratio under average temperature and pressure to remove or decompose organic substances in wastewater [16]. Among these procedures, the Fenton method causes numerous interests due to its convenience and effectiveness. Notably, the Fenton method can produce many hydroxyl radicals (∙OH) by introducing divalent iron solution and hydrogen peroxide, as shown in Equation 1 below.


[1] $${\text{Fe}}^{2+}+{\text{H}}_{2}{\text{O}}_{2}\to {\text{Fe}}^{3+}+{\text{OH}}^{-}+{\text{OH}}^{\cdot }$$

The Fenton method exhibits high oxidation capability and low selectivity for removing most organic substances. It can decompose organic pollutants into smaller organic molecules and generate carbon dioxide, water, and inorganic ions [17]. Generally, the ferrous ion employed in the Fenton reaction is from ferrous sulfate (FeSO_4_), which can provide a uniform reaction system due to its high solubility. Unfortunately, it might cause two severe shortcomings in the Fenton reaction process. First, ferric ions (Fe^3+^) formed in the Fenton reaction will interact with the excess hydroxide ion (OH^−^) to produce Fe(OH)_3_ precipitation, which is called iron sludge. It might decrease the activity and lead to the termination of the Fenton reaction. Second, ferric ions might easily cause complicated chain reactions with organic matters, resulting in the formation of Fe^3+^ complexes or other intermediate products. Such complexes might compete with the hydroxyl radicals, eliciting a degradation of the reaction performance [18].

In recent years, the Fenton method has gradually developed into a new scenario of oxidation method, called photo-Fenton, which is facilitated or driven by the light source. Compared with a typical Fenton reaction, a photo-Fenton reaction excited by ultraviolet light or visible light can achieve a faster reaction rate and a complete degree of oxidation [19]. Besides, it shows a positive relationship between light intensity and photocatalytic activity. With the assistance of light irradiation, the hydrogen peroxide can be remarkably transformed into redox radicals, followed by destroying the organic pollutants. Meanwhile, the remaining divalent iron complexes in the system can return to the circulation of hydrogen peroxide reaction and continuously form new hydroxide radicals [20]. Therefore, based on our knowledge of photocatalysis [21–22], the benefits of Fenton reaction and photocatalysis are combined to develop a conceptual catalytic design to expand the photocatalytic pathway of Fenton oxidation, called photocatalytic Fenton-like reaction or heterogeneous photo-Fenton-like reaction [23].

LaFeO_3_ perovskite oxides are promising materials to conduct Fenton-like oxidation to decompose organic pollutants with light irradiation. Some literature exhibits the capability of LaFeO_3_ perovskite oxides as photocatalysts to degrade organic contaminants. Li et al. prepared intrinsic LaFeO_3_ or SmFeO_3_ nanoparticles via the sol–gel method to decompose rhodamine-B under visible light irradiation. With the assistance of H_2_O_2_, it shows a synergistic effect between photocatalytic reaction and heterogeneous photo-Fenton-like reaction [23]. Furthermore, the strategies of being loaded over supports (such as g-C_3_N_4_ [24], carbon spheres [25], BiOBr [26], and Ag_2_CrO_4_ [27]) to form heterojunction structures or doping other atoms into LaFeO_3_ [28] are comprehensively developed. For instance, Orak et al. impregnated LaFeO_3_ or LaTi_0.15_Fe_0.85_O_3_ on the monolithic cordierite structure, which could provide light transmittance and suitable surface area for degrading methylparaben. Although Ti-doped catalyst was expected as a semiconductor to enhance the photocatalytic efficiency, pure LaFeO_3_ still revealed the better performance of methylparaben photodegradation than LaTi_0.15_Fe_0.85_O_3_ [28]. On the contrary, Garcia-Muñoz et al. attempted to substitute Ti to Fe within LaFeO_3_ as the mediator of heterogeneous Fenton-like reaction to remove 4-chlorophenol in water. The network with Ti substitution (Ti/Fe molar ratio = 0.21) provided stronger robustness, resulting in lower iron release and higher activity enhancement [29]. Ti-doped, Mn-doped [30], and Cu-doped [31] LaFeO_3_ were investigated to conduct a photocatalytic Fenton-like reaction. Jauhar et al. demonstrated that Mn substituting Fe within LaFeO_3_ with the molar ratio of 0.1–0.5 displayed the effect of being used as a heterogeneous photocatalyst for dye degradation. Though it was proven that Mn-doped LaFeO_3_ could enhance the activity of heterogeneous photo-Fenton-like reactions, the influence of Mn content on the activity was not significant [30]. Phan et al. verified that Cu-doped LaFeO_3_ exhibited physicochemical properties to decolorize methlyorgane, methylene blue, and rhodamine B under visible light irradiation. Their optimal sample was LaFe_0.85_Cu_0.15_O_3_, which could remove dyes much more efficiently due to more generation of hydroxyl radicals than pure LaFeO_3_ [31].

Ni-doped LaFeO_3_ was ubiquitously employed as a photo/thermal catalyst or a catalyst precursor for VOCs combustion [32], hydrogen production from ethanol [33], hydrocarbon fuels production from CO_2_ and H_2_O [34], syngas production from dry reforming [35], steam reforming of methane [36], or combined reforming of methane with CO_2_ and O_2_ [37]. Meanwhile, LaNiO_3_ photocatalysts also played an essential role in photocatalytic reactions for wastewater [38]. Fe doping of LaNiO_3_ revealed the potential of tuning bandgap and boosting the light absorption to degrade RhB [39]. However, little literature comprehensively and systematically discusses the effect of different doping ratios on photocatalytic reactions. Moreover, LaNiO_3_ revealed broad absorption in the visible light range [38], so the Ni doping was expected to improve the visible light harvesting of LaFeO_3_. Accordingly, little literature explored the effect of Ni substitution to LaFeO_3_ on the performance of photocatalytic Fenton-like reaction to degrade dye pollutants in water. Therefore, in this study, various contents of Ni-doped LaFeO_3_ were examined to remove organic pollutants under simulated solar light irradiation. Methylene blue is the representative compound of thiazine dyes and tetracycline is the indicator pollutant to represent the antibiotics of PPCPs accordinly.

## Results and Discussion

### Material characterization of various photocatalysts

**Figure 1:**
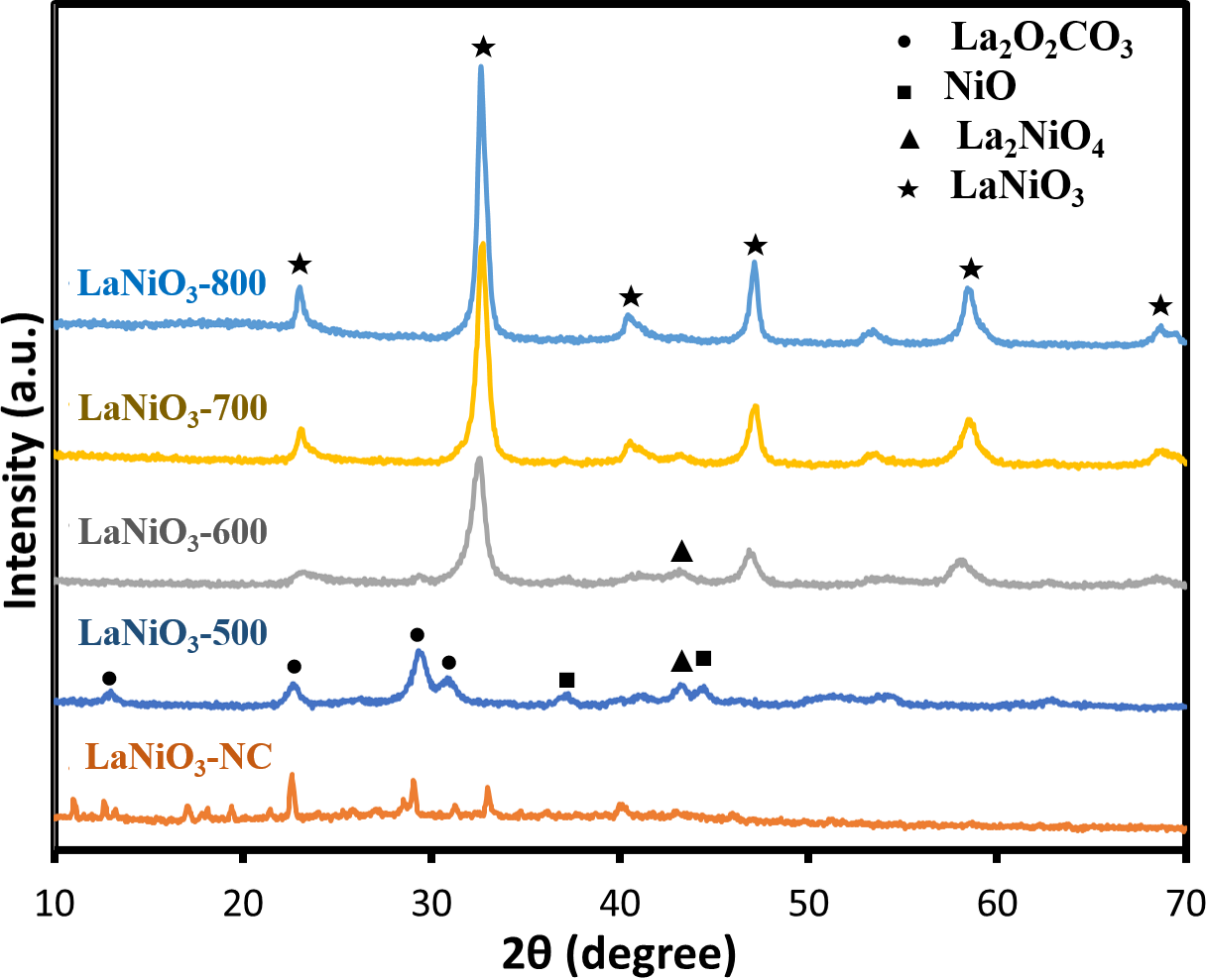
The characteristic XRD patterns of LaNiO_3_ at different calcination temperatures.

X-ray powder diffraction (XRD) was used to reveal the structure of the materials. In the synthesis step of LaNiO_3_, the calcination temperature was set to 500, 600, 700, and 800 °C, respectively, and the samples were named LaNiO_3_-500, LaNiO_3_-600, LaNiO_3_-700, LaNiO_3_-800 in sequence. The uncalcined sample was noted as LaNiO_3_-NC. As shown in Figure 1, there was no crystalline LaNiO_3_ signal for the sample of LaNiO_3_-NC, but there were other signals for other samples indicating the presence of La_2_NiO_4_ (42.8°, JCPDS Card #011-0557), NiO (37.3°, 43.3°, JCPDS Card #04-0835), and La_2_O_2_CO_3_ (13.1°, 22.8°, 29.6°, 31.3°, JCPDS Card #23-0322) signals, respectively [40]. The sample calcined at 500 °C exhibited the more apparent signals belonging to La_2_O_2_CO_3_ and NiO. The signal of LaNiO_3_ did not appear for LaNiO_3_-500, indicating that the temperature of 500 °C was not enough to form LaNiO_3_. With the increase of temperature up to 600 °C, some obvious 2θ signals of LaNiO_3_ crystalline appeared, including 23.3°, 32.8°, 41.2°, 47.3°, 58.6°, and 68.8°. These peaks indicated the crystal planes (012), (110), (202), (024), (211), and (220) of LaNiO_3_, respectively, conforming to JCPDS Card #033-0711 [41]. LaNiO_3_-700 and LaNiO_3_-800 performed similar peak positions with higher intensity of signals. Accordingly, the crystal diameter of LaNiO_3_ was calculated by Scherrer's equation [42]. The crystal diameters of LaNiO_3_-600, LaNiO_3_-700, and LaNiO_3_-800 were 8.5 nm, 11.9 nm, and 14.6 nm, respectively. It could be seen that the higher the calcination temperature, the higher the crystal size will form.

**Figure 2:**
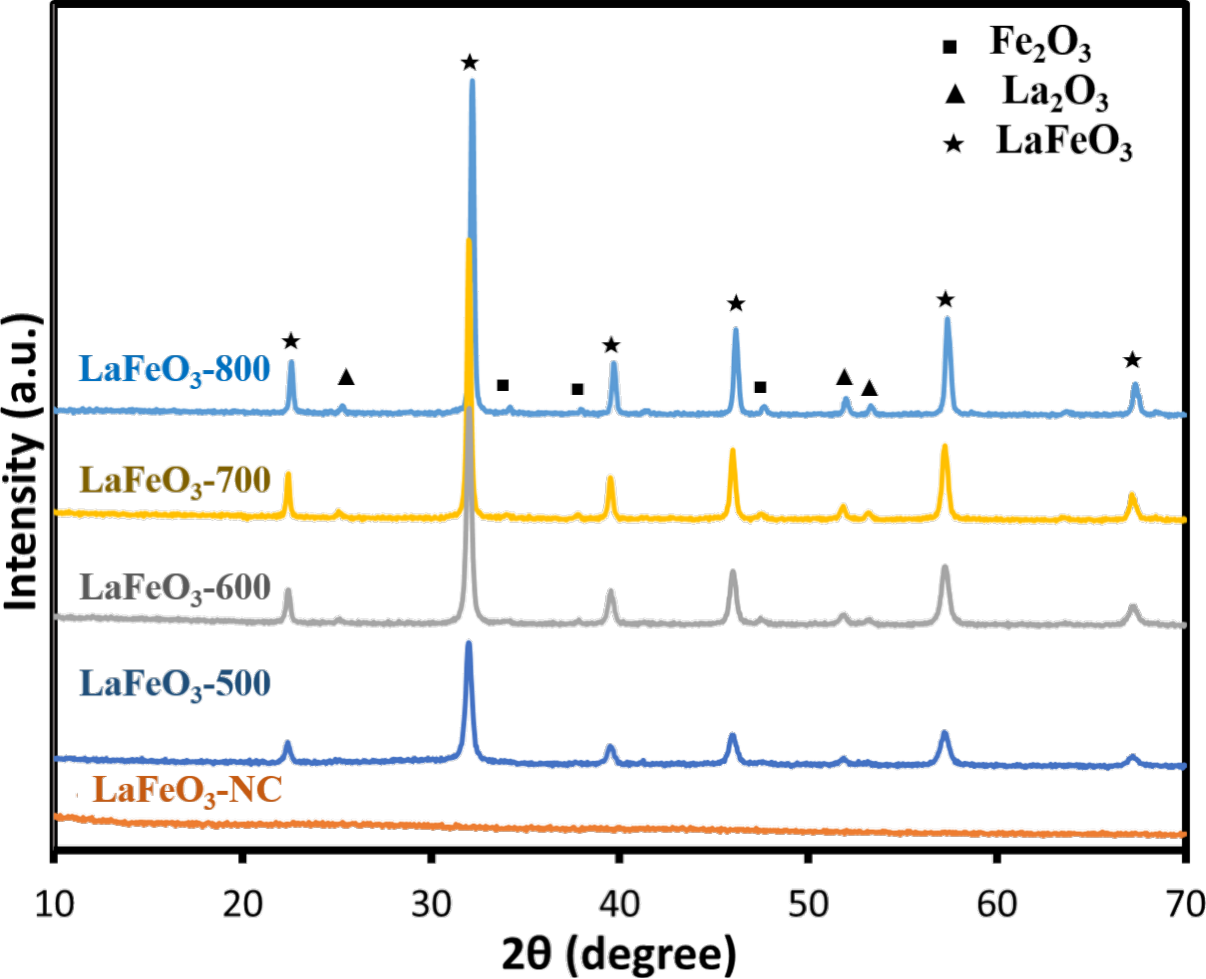
The characteristic XRD patterns of LaFeO_3_ at different calcination temperatures.

Similarly, LaFeO_3_ samples with different calcination temperatures of uncalcined, 500, 600, 700, and 800 °C were noted as LaFeO_3_-NC, LaFeO_3_-500, LaFeO_3_-600, LaFeO_3_-700, and LaFeO_3_-800, respectively. In Figure 2, there was no signal for the uncalcined sample. While the calcination temperature was set at 500 °C, the signals of the LaFeO_3_ crystalline phase appeared. The diffraction peak of LaFeO_3_ became stronger as the calcination temperature increased. As shown in Figure 2, the 2θ peaks of 22.6°, 32.2°, 39.6, 46.3°, 57.4°, and 67.4° indicated the crystal planes of (101), (121), (220), (202), (240), and (242), according to JCPDS Card #037-1493 [43]. With the increase in calcination temperature, the higher crystal diameters of LaFeO_3_ calculated by Scherrer's equation were obtained. The crystal diameters was 18.5 nm, 25.4 nm, 29.1 nm, 35.0 nm for LaFeO_3_-500, LaFeO_3_-600, LaFeO_3_-700, LaFeO_3_-800, respectively, suggesting higher calcination temperature caused higher crystallinity for LaFeO_3_. Moreover, all LaFeO_3_ samples revealed higher crystal diameters than LaNiO_3_, indicating LaFeO_3_ tended to grow crystal than LaNiO_3_ at a certain calcination temperature. However, high temperature might cause particle aggregation, leading to the lower surface area. Therefore, a moderate temperature of 700 °C was selected to obtain the perovskite materials. On the other hand, the XRD of LaFeO_3_-800 indicated the appearance of Fe_2_O_3_ at 2θ peaks of 32.9°, 38.3°, 47.3° (JCPDS Card #39-0238) and La_2_O_3_ at 2θ peaks of 25.3°, 52.0°, 54.1° (JCPDS Card #40-1281). Especially, the peak of 32.9° for LaFeO_3_-800 was much clearer than that of LaFeO_3_-700, suggesting that the calcination temperature was too high and caused the formation of Fe_2_O_3_.

**Figure 3:**
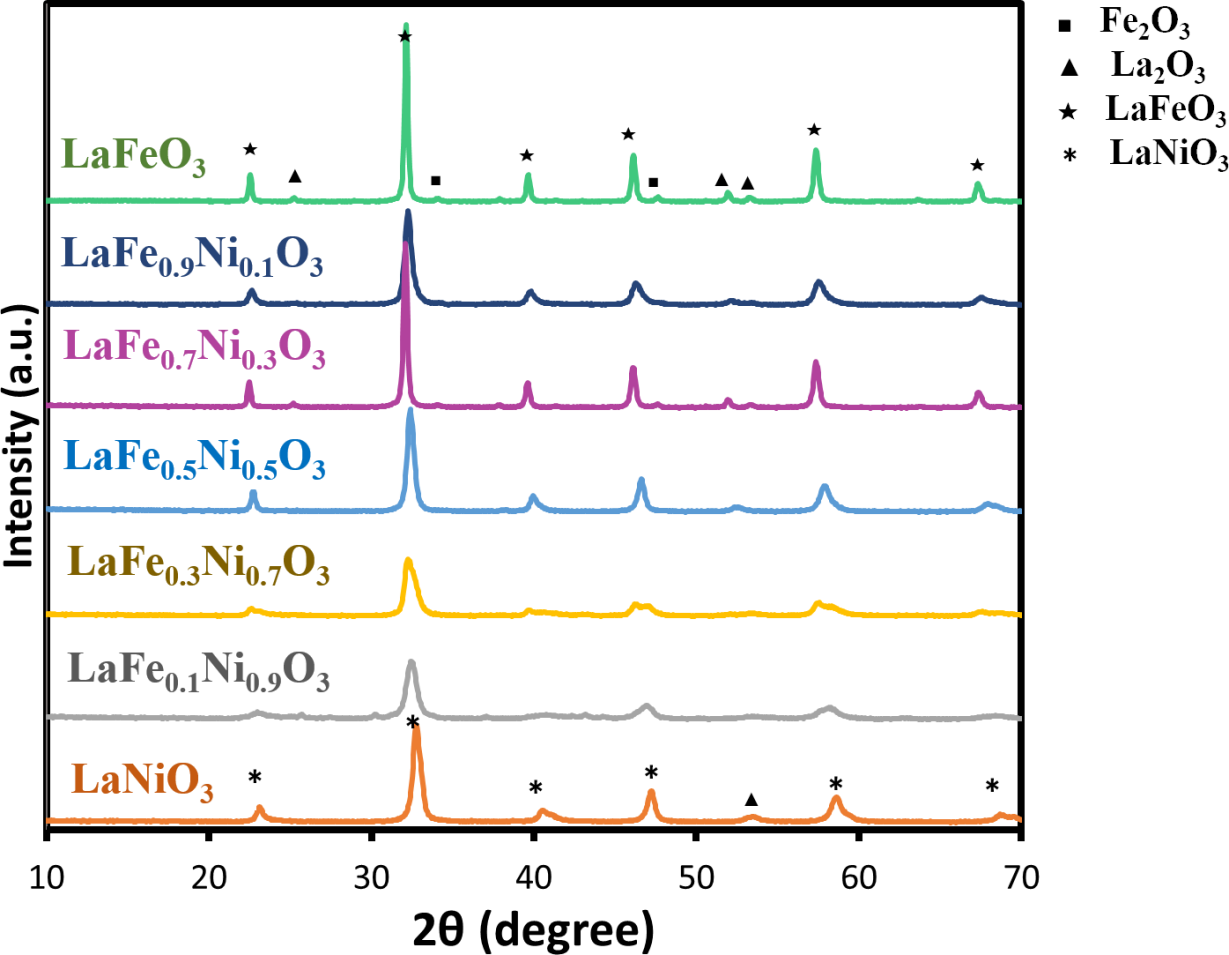
The characteristic XRD patterns of LaFe*_x_*Ni_1−_*_x_*O_3_.

**Figure 4:**
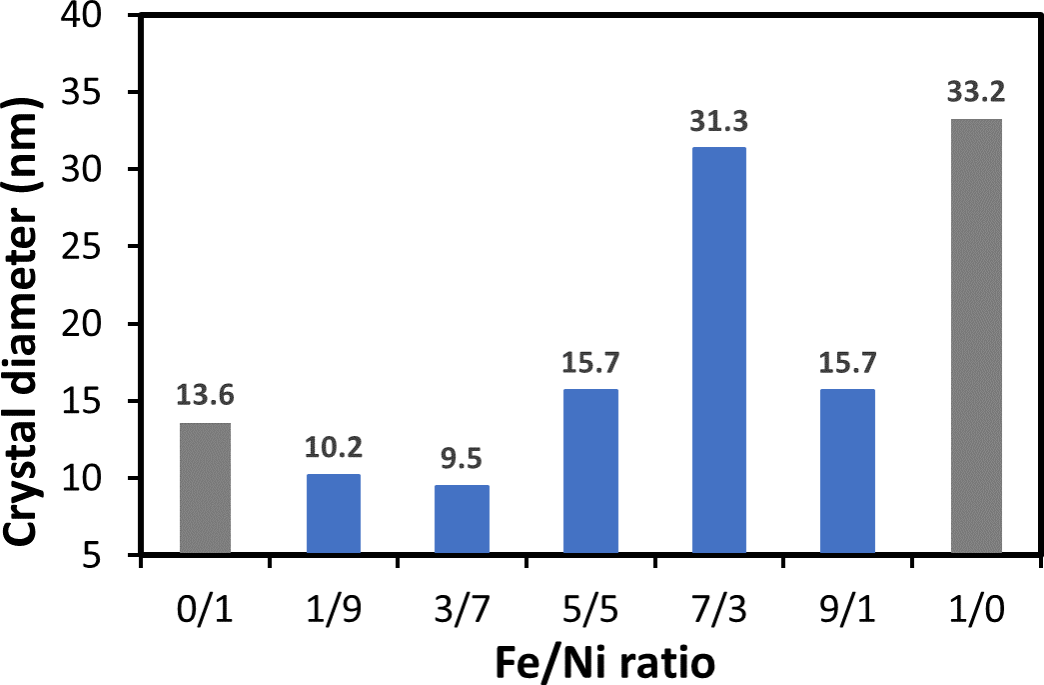
The crystal diameters of samples with various Fe/Ni doping ratios.

In addition, samples with various doping proportions of Fe/Ni, including 0.1/0.9, 0.3/0.7, 0.5/0.5, 0.7/0.3, and 0.9/0.1 were synthesized. The pH value was adjusted at 0 during synthesis, and the calcination temperature was fixed at 700 °C. The samples were named LaFeO_3_, LaFe_0.9_Ni_0.1_O_3_, LaFe_0.7_Ni_0.3_O_3_, LaFe_0.5_Ni_0.5_O_3_, LaFe_0.3_Ni_0.7_O_3_, LaFe_0.1_Ni_0.9_O_3_, LaNiO_3_ to represent the different Fe/Ni doping ratios. In Figure 3, the signals of LaFeO_3_ and LaNiO_3_ were identified by JCPDS Card #037-1493 [43] and JCPDS Card #033-0711 [41], respectively. Among these peaks, the main characteristic peak around 32° was slightly shifted for different Fe/Ni doping ratios, indicating that Fe and Ni were successfully doped into the structure of perovskite oxides. It is interesting to note that LaFe_0.7_Ni_0.3_O_3_ had a stronger signal than other doped samples. The crystal diameters of samples with various Fe/Ni doping ratios were also calculated by Scherrer's equation, as shown in Figure 4, which ranged from 9.5 to 31.3 nm. When the Fe/Ni ratio was manipulated at 7/3, the sample had the largest crystal diameter of 31.3 nm. The better crystallinity caused less recombination of electron-and-hole pairs, and subsequent reactions might occur more effectively [44]. On the other hand, The unit cell parameters and cell volume were also estimated from the XRD patterns and summarized in Supporting Information File 1, Table S2. Since the peaks of LaFe_0.5_Ni_0.5_O_3_, LaFe_0.7_Ni_0.3_O_3_, and LaFe_0.9_Ni_0.1_O_3_ were closed to LaFeO_3_, the unit cell parameters and interplanar spacing were calculated based on the model of orthorhombic LaFeO_3_ (JCPDS card: 037-1493) using Bragg’s law [45]. Meanwhile, LaFe_0.5_Ni_0.5_O_3_, LaFe_0.3_Ni_0.7_O_3_, and LaFe_0.1_Ni_0.9_O_3_ were also calculated based on the model of hexagonal LaNiO_3_. As a result, the lattice constant and cell volume were slightly expanded. It was interesting to note that LaFe_0.7_Ni_0.3_O_3_ revealed a relatively larger expansion, which had a higher cell volume, suggesting a better separation of photo-induced electron and hole pairs.

**Figure 5:**
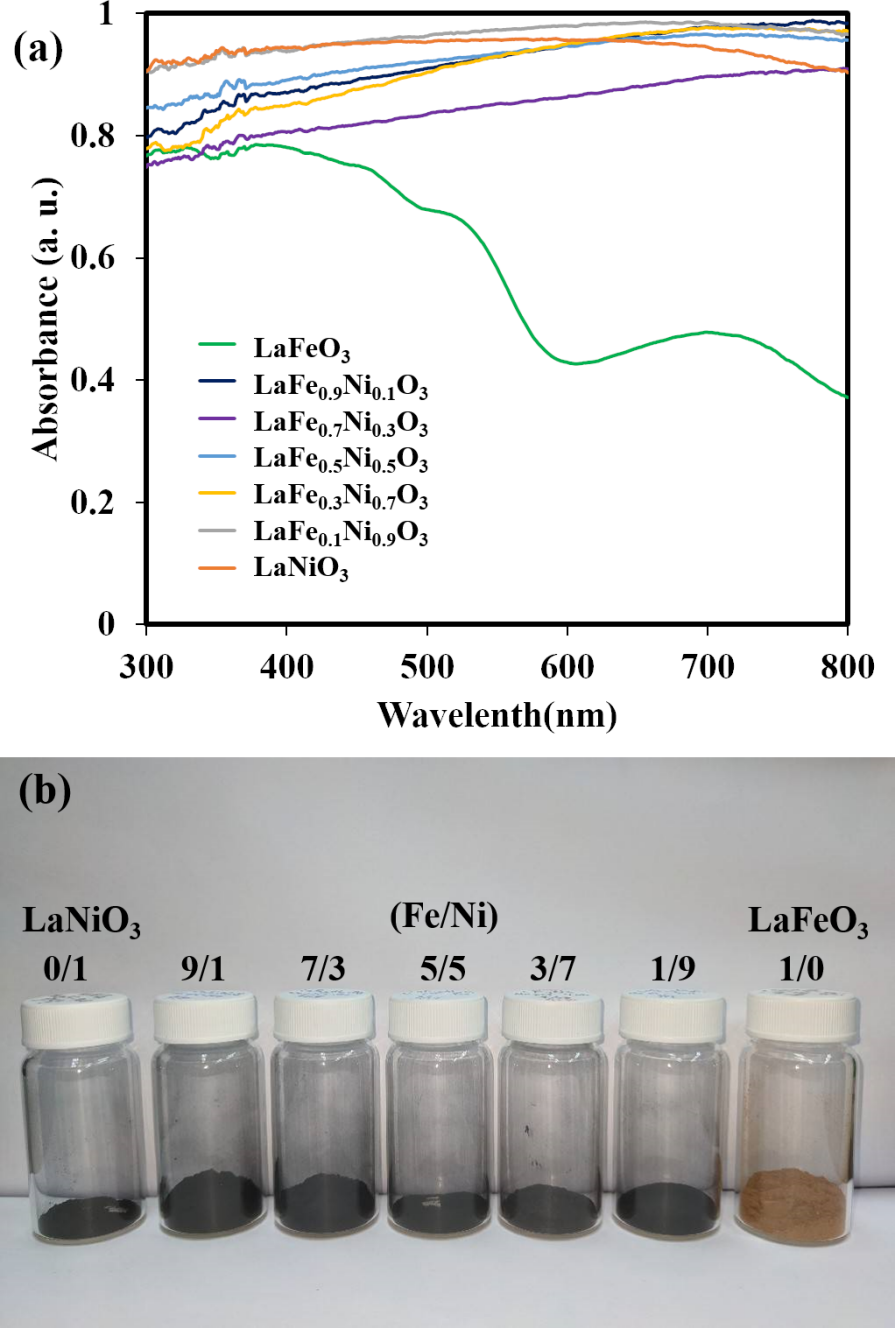
(a) The DRS spectrum and (b) the pictures of samples with various Fe/Ni doping ratios.

The UV–vis absorption capability with diffuse reflectance spectroscopy (DRS) and photographs of various LaFe*_x_*Ni_1−_*_x_*O_3_ perovskite oxides with different proportions were shown in Figure 5a and 5b. Except for LaFeO_3,_ which was brown, the rest of the perovskite oxides doped with Ni became black. Since pristine LaNiO_3_ was black, it exhibited the total absorption in the ultraviolet–visible light spectrum, consistent with the literature comparison [46]. For comparison, LaFeO_3_ revealed an apparent absorption shoulder between 500 and 600 nm in Figure 5a, similar to the previous study [47]. To enhance the light absorption of LaFeO_3_, it was an effective method to dope Ni into the perovskite oxides. Accordingly, the samples were doped with Ni to form LaFe*_x_*Ni_1−_*_x_*O_3_ perovskite oxides that could absorb the most visible and ultraviolet light spectrum. Thus, the prepared Ni-doped LaFeO_3_ perovskite oxides were presented as black, as shown in Figure 5b. They successfully increased the absorption efficiency of visible light and utilized more visible light effectively.

**Figure 6:**
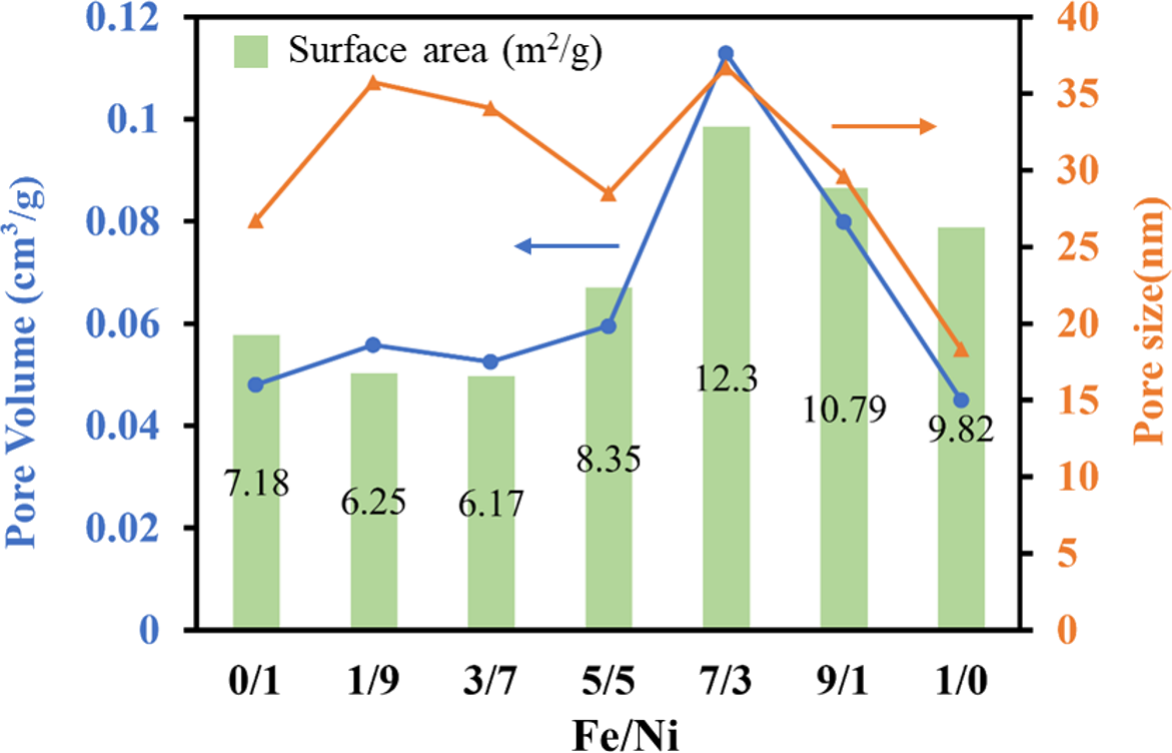
Specific surface area, pore size, and pore volume of the samples with different Fe/Ni ratios.

In order to determine the specific surface area, pore size, and pore volume of the prepared perovskite oxides, the analysis of nitrogen adsorption and desorption was performed. As shown in Supporting Information File 1, Figure S1, all LaFe*_x_*Ni_1−_*_x_*O_3_ perovskite oxides were in line with Type IV isotherm according to their hysteresis phenomenon. The Brunauer–Emmett–Teller (BET) result of the pristine LaFeO_3_ and LaNiO_3_ corresponded to the result in the literature [48–49]. The pore size distribution of the samples was shown in Figure S2. The distribution between 2 and 50 nm indicated that the prepared perovskite oxides were mesoporous. The summary of the specific surface area, pore size, and pore volume for all the samples with different Fe/Ni ratios was presented in Table S1. In Figure 6, it could be found that the LaFe_0.7_Ni_0.3_O_3_ with the Fe/Ni ratio of 7/3 had the highest specific surface area, pore volume, and pore size, suggesting there was more possibility for LaFe_0.7_Ni_0.3_O_3_ to adsorb and react with the molecules on the surface. On the other hand, the prepared samples might also be considered non-porous materials with inter-particle pore voids, since their low surface area might come from the external surface, indicating that LaFe_0.7_Ni_0.3_O_3_ had the highest external surface area for reaction.

**Figure 7:**
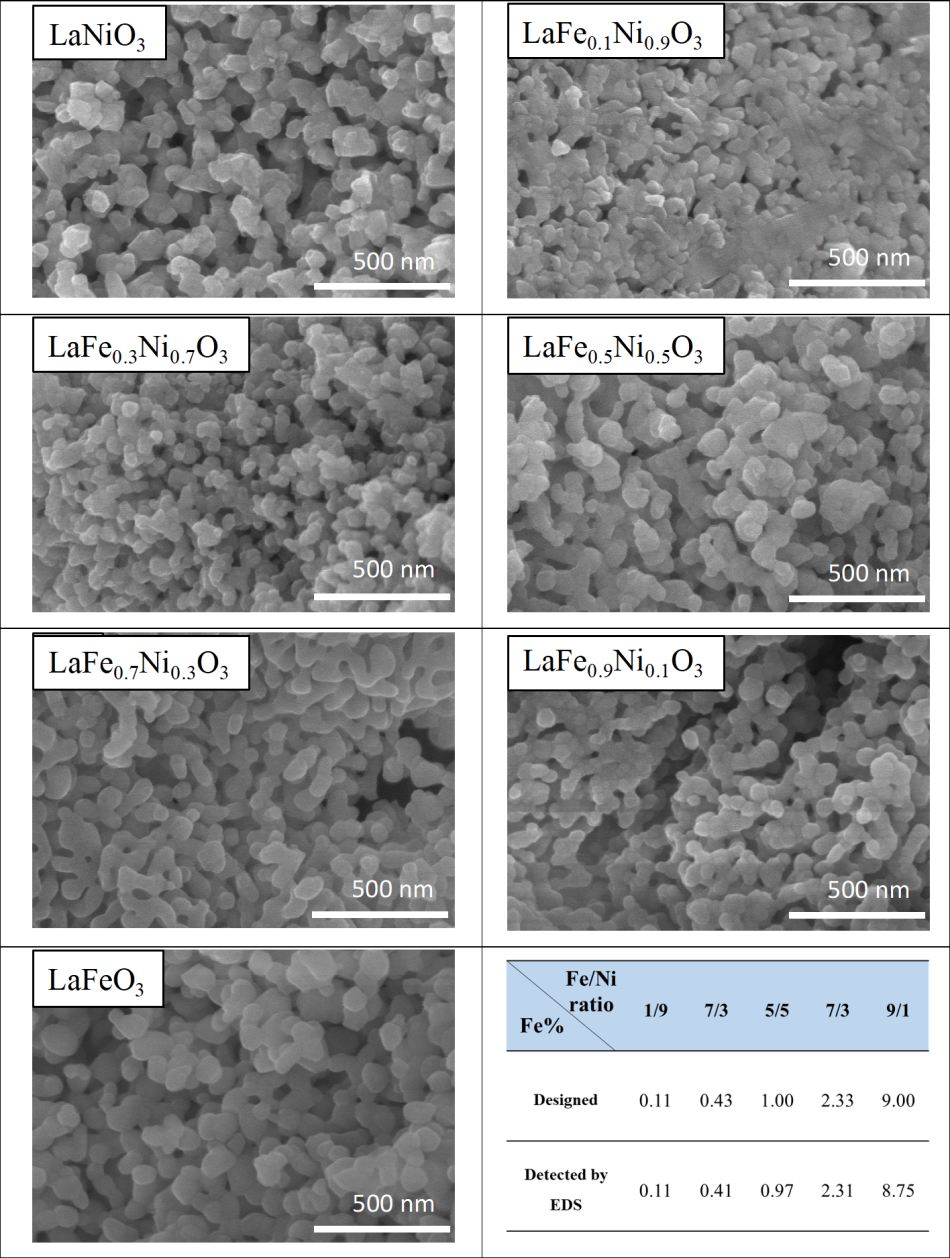
The FESEM images of LaFe*_x_*Ni_1−_*_x_*O_3_ prepared at pH 0 (at the magnification of 100,000×).

Figure 7 revealed the field emission scanning electron microscopy (FESEM) images at the magnification of 100,000×, the surface of the samples with different Fe/Ni ratios were irregular and slightly different from each other. The grain surfaces of the pure LaNiO_3_ and LaFeO_3_ were chestnut-like. The observed appearance was also similar to the literature situation [50]. With the increased Fe content, some small lumps formed on the surface. When Fe/Ni ratio reached 7/3, relatively abundant small particles were generated than other samples. Small particles of LaFe_0.7_Ni_0.3_O_3_ would increase the surface area, which was consistent with the trend of the results detected by BET. On the other hand, it is interesting to note that the pH value during synthesis could affect the appearance of LaFe*_x_*Ni_1−_*_x_*O_3_. The samples prepared at pH 0 showed more uniform than that at pH 7 (shown in Supporting Information File 1, Figure S3). It indicated that the protons in the sol–gel solution could help the separation of LaFe*_x_*Ni_1−_*_x_*O_3_ crystals, leading to less particle aggregation. Moreover, the elemental analysis of the samples was also carried out using energy dispersive spectroscopic (EDS). The Fe contents of the samples with different Fe/Ni atomic ratios were identified. EDS detection showed that the synthesized samples exhibited the accurate Fe/Ni atomic ratios as designed. The detailed EDS data was provided in Table S3. The lanthanum, nickel, iron, and oxygen were analyzed from the samples, and the carbon was detected from the carbon tape.

### MB removal test using various photocatalysts prepared at pH 0 or pH 7

**Figure 8:**
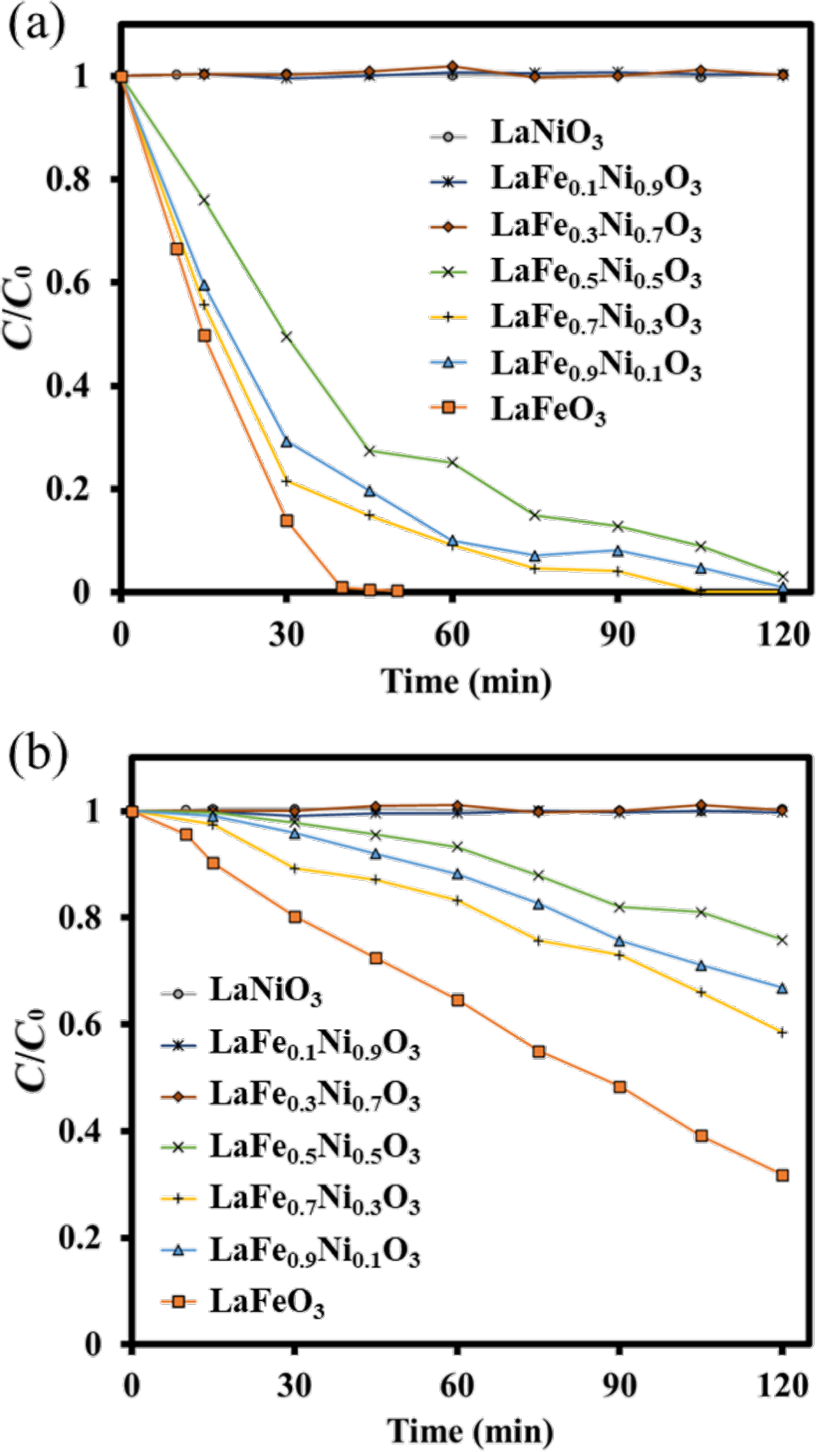
MB degradation experiments using various LaFe*_x_*Ni_1−_*_x_*O_3_ with different Fe/Ni ratios prepared at (a) pH 0; (b) pH 7.

The standard concentration of MB aqueous solution was prepared at 20 ppm. Various LaFe*_x_*Ni_1−_*_x_*O_3_ perovskite oxides prepared at pH 0 were examined for dark adsorption and photocatalytic degradation. First, dark adsorption was not significant for all perovskite oxides since their specific surface areas were low. After 30 min, to confirm the achievement of dark adsorption-desorption equilibrium, the photocatalytic reaction occurred under the irradiation of simulated AM 1.5G solar light with adding H_2_O_2_. By monitoring the *C*/*C*_0_ of MB, the performance of all perovskite oxides prepared at pH 0 or pH 7 were depicted in Figure 8a and 8b, respectively. LaFeO_3_ had the highest content of Fe^3+^ ions so that it could generate more hydroxyl radicals with H_2_O_2_ in the solution, resulting in the best performance of MB degradation. LaFeO_3_ could completely degrade MB after 45 min of simulated solar light irradiation due to the Fenton effect of Fe^3+^ [51]. On the contrary, LaNiO_3_ did not conduct the Fenton-like effect; therefore, it exhibited poor photocatalytic ability. Fe's phenomenon revealed better catalytic activity than Ni, similar to the previous study [52]. It might result from Ni's apparent activation energy being higher than Fe's for producing oxidizing species [53]. Although Fe was attempted to be doped into LaNiO_3_, LaFe_0.1_Ni_0.9_O_3_ and LaFe_0.3_Ni_0.7_O_3_ still exhibited low photocatalytic capability. Until Fe doped amount was up to 50% for replacing Ni, the photocatalytic performance of LaFe_0.5_Ni_0.5_O_3_ was enhanced much more apparent than that of LaFe_0.3_Ni_0.7_O_3_. It indicated that Fe^3+^ plays a vital role in involving MB degradation. As the Fe doped content increased, LaFe_0.7_Ni_0.3_O_3_ reached the highest photocatalytic capability, originating from its largest surface area and crystal diameters. A larger surface area would enhance the surface reaction with the aqueous solution, and larger crystal diameters could decrease the possibility of recombining electron-and-hole pairs. However, while the Fe doped content was set at 90%, replacing Ni, the photocatalytic capability was reduced due to the lower surface area and smaller pore size. The above inference was consistent with the cases of the samples prepared at pH 0 and pH 7. Interestingly, the performance of the perovskite oxide prepared at pH 0 was much better than that prepared at pH 7. It was derived from the fact that more uniform structural features of the perovskite oxide prepared at pH 0 were achieved than the condition at pH 7. Moreover, the photocatalytic performance of physically mixed 70% LaFeO_3_ and 30% LaNiO_3_ could be estimated based on the result of MB degradation using 70% LaFeO_3_ (since LaNiO_3_ showed no MB degradation). The degradation of 70% LaFeO_3_ in 30 min was approximately 60.0%. Considering the light shading effect by LaNiO_3_, the MB photodegradation of physically mixed 70% LaFeO_3_ and 30% LaNiO_3_ might be lower. On the contrary, the LaFe_0.7_Ni_0.3_O_3_ showed 78.5% of MB degradation in 30 min. Therefore, we believed there would be a benefit of doping Ni to improve the photodegradation.

**Figure 9:**
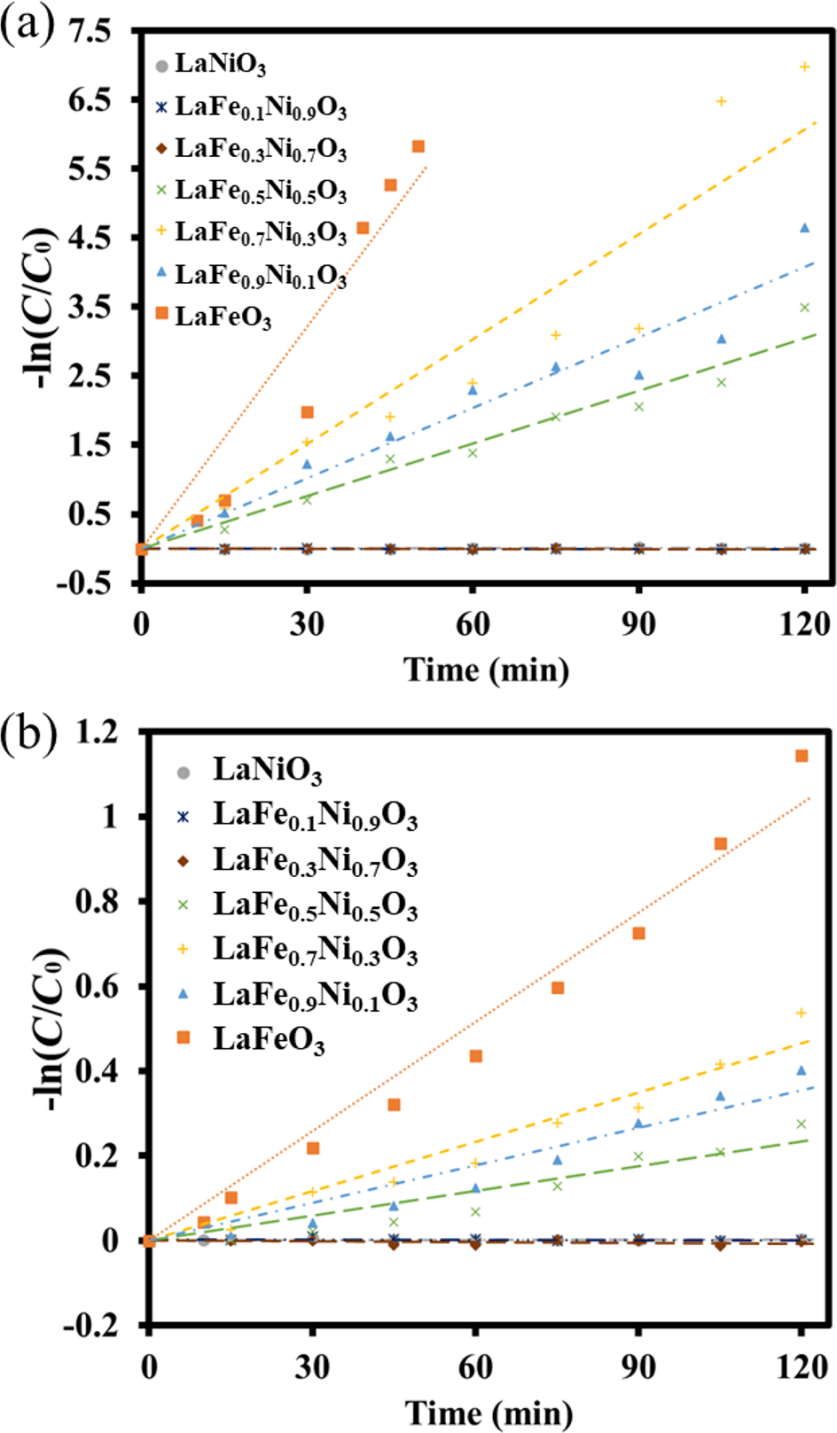
Kinetic analysis of MB degradation experiments using various LaFe*_x_*Ni_1−_*_x_*O_3_ with different Fe/Ni ratios at (a) pH 0 and (b) pH 7.

In Figure 9, by taking the negative natural logarithmic value of *C*/*C*_0_, it was observed that all the degradation trends were in line with first-order kinetics:


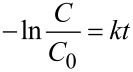


where *k* was the rate constants of MB degradation reaction; *t* was the reaction duration in min. As shown in Table 1, for photocatalytic Fenton-like reaction to decompose MB dye, LaFeO_3_ had the fastest degradation rate (*k* = 0.1072 prepared at pH 0; *k* = 0.0086 prepared at pH 7). It might be due to the higher content of Fe ion for LaFeO_3_ than LaFe_0.7_Ni_0.3_O_3_. However, we would like to focus on the doping effect on photocatalytic reaction so that Fe_0.7_Ni_0.3_O_3_ was the target material to be further analyzed and characterized. Comparing the samples co-doped with Fe and Ni, LaFe_0.7_Ni_0.3_O_3_ exhibited a higher *k* value of 1st order reaction than other co-doped samples. LaFe_0.7_Ni_0.3_O_3_ had a larger crystal diameter and higher specific surface, which improved the separation of photogenerated charge carriers and the efficiency of the surface reaction. For comparison, the second-order kinetics analysis was also conducted for the samples prepared at pH 0 in Table 1. However, the R^2^ values were too low to represent their kinetic model. Therefore, 1st order reaction kinetics was more suitable for describing the kinetic model of LaFe*_x_*Ni_1−_*_x_*O_3_.

**Table 1:** The kinetic analysis of LaFe*_x_*Ni_1−_*_x_*O_3_ perovskite oxides prepared at pH 0 and pH 7.

pH value	Reaction kinetics analysis		LaFeO_3_	LaFe_0.9_Ni_0.1_O_3_	LaFe_0.7_Ni_0.3_O_3_	LaFe_0.5_Ni_0.5_O_3_

pH 0	1st order	*k*	0.1072	0.0339	0.0506	0.0254
		R^2^	0.9616	0.9808	0.9584	0.9876
	2nd order	*k*	0.8043	0.0313	0.4098	0.0125
		R^2^	0.6892	0.4846	0.5404	0.6184
pH 7	1st order	*k*	0.0086	0.003	0.0039	0.002
		R^2^	0.9903	0.9691	0.9838	0.9517

### MB removal test under different conditions using LaFe_0.7_Ni_0.3_O_3_ prepared at pH 0

**Figure 10:**
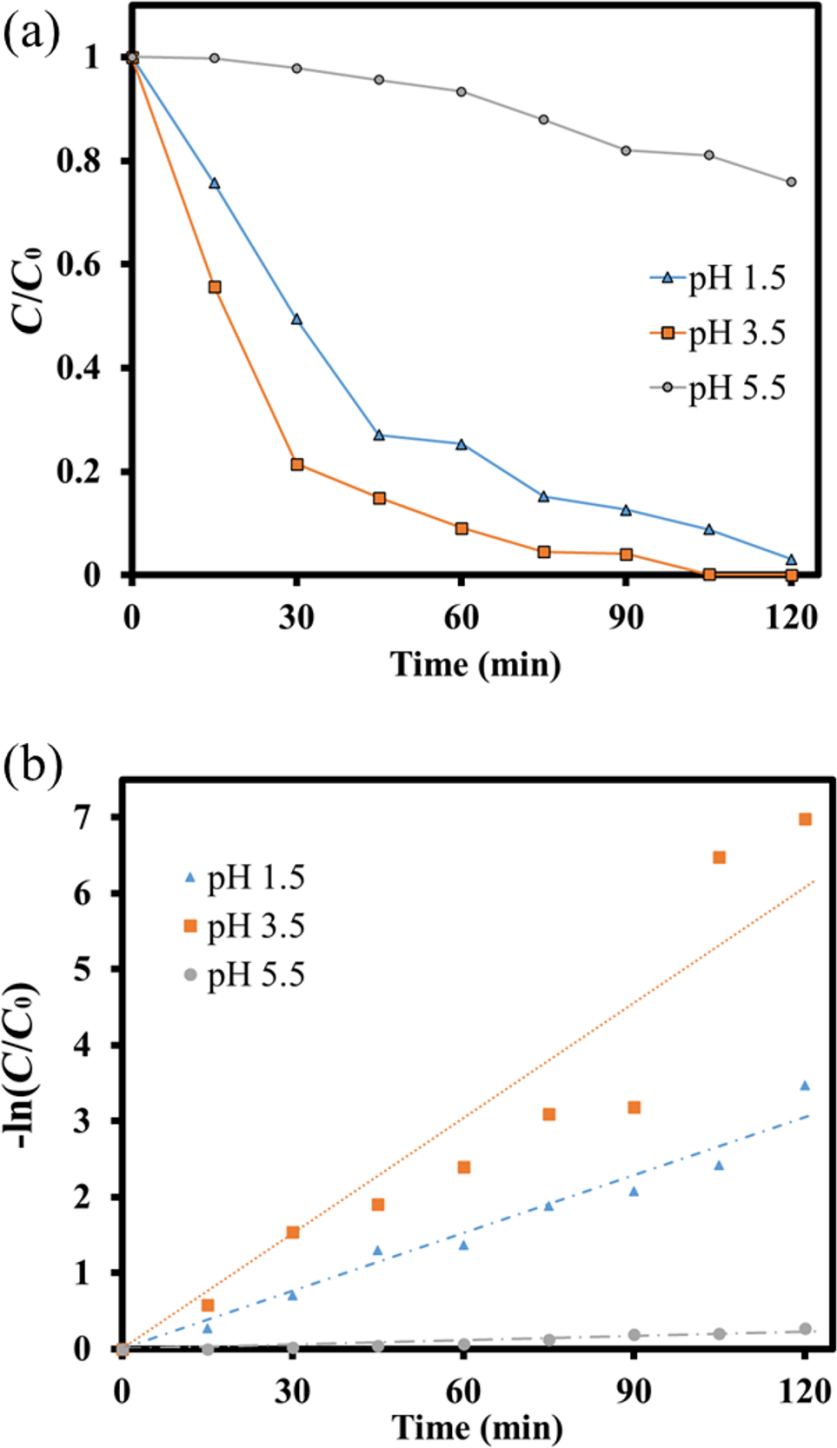
(a) The *C*/*C*_0_ and (b) 1st order kinetic analysis of the MB photodegradation using LaFe_0.7_Ni_0.3_O_3_ operating at different pH values.

The pH value of the solution was a strong effect on photocatalytic degradation [54]. Accordingly, different pH values of solution using LaFe_0.7_Ni_0.3_O_3_ perovskite oxides prepared at pH 0 were examined for photocatalytic degradation. Thus, the MB aqueous solution was adjusted to pH 1.5, 3.5, and 5.5. The performance of the photocatalytic Fenton degradation was measured at different pH values in Figure 10. After 120 min of simulated solar light irradiation, the degradation performance at 1.5, 3.5, and 5.5 were 97.9%, 100%, and 25.2%, respectively. The pH value at 3.5 revealed the highest photocatalytic performance, in which MB pollutants were completely degraded within 105 min. Based on the 1st order kinetic analysis, the reaction rate constants (*k*) at pH 1.5, 3.5, and 5.5 were 0.0254, 0.0506, and 0.002, respectively. While the pH value was too high, the hydrogen peroxide in the solution was easily decomposed into oxygen and water [55]. On the other hand, when the pH value was too low, the solution tended to generate too many hydrogen ions, which would react with hydrogen peroxide to form water, leading to a decrease in the photocatalytic degradation performance [56]. Therefore, the operating condition of the pH value was set at 3.5.

**Figure 11:**
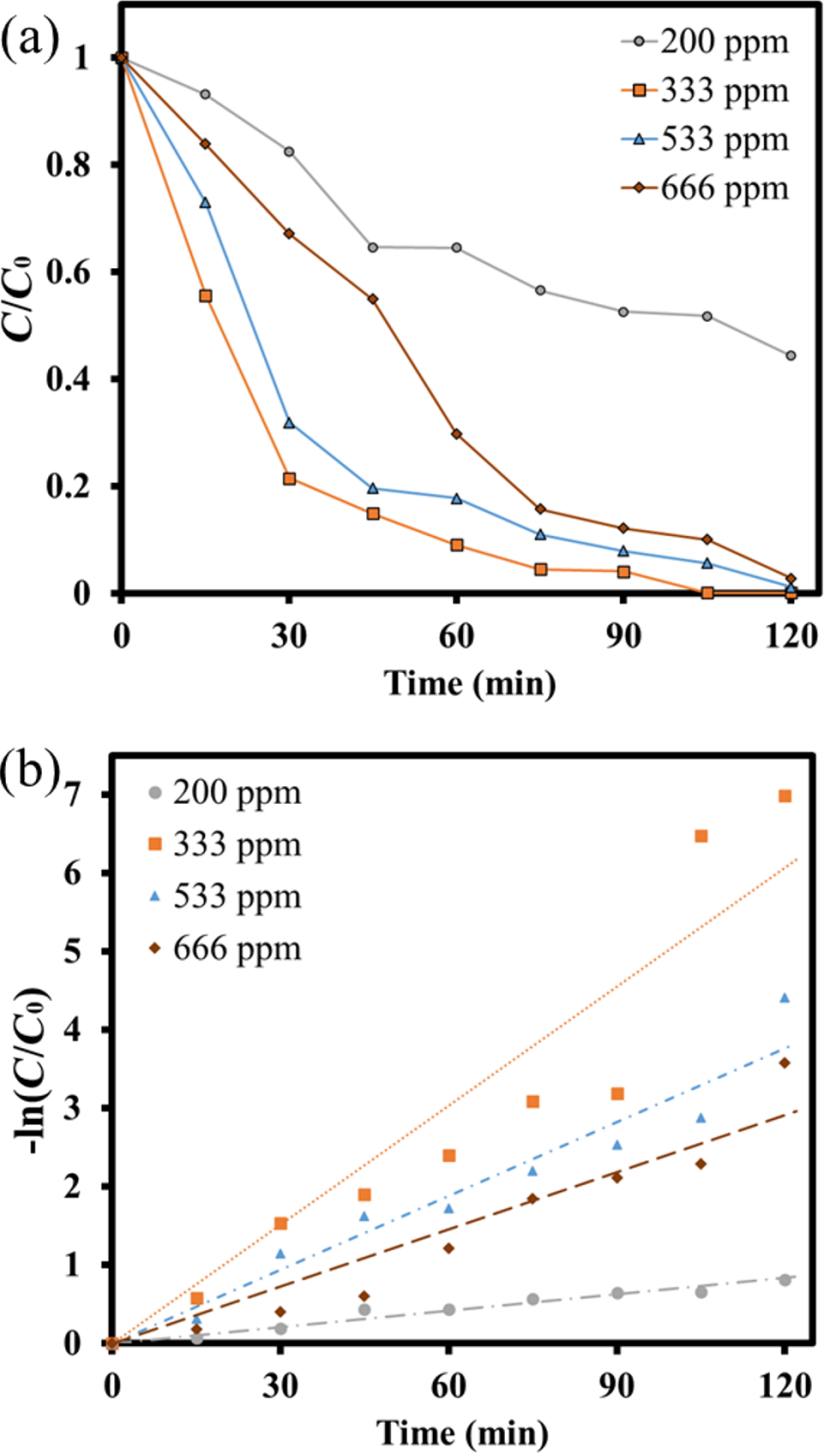
(a) The *C*/*C*_0_ and (b) 1st order kinetic analysis of the MB photodegradation using LaFe_0.7_Ni_0.3_O_3_ operating at different catalyst dosages.

After manipulating the operating pH value, the pH of the solution was fixed at 3.5. The dosages of LaFe_0.7_Ni_0.3_O_3_ perovskite oxides were further examined. In this study, the amount of the MB solution was 150 mL, and various LaFe_0.7_Ni_0.3_O_3_ perovskite oxides, including 30 mg (200 ppm), 50 mg (333 ppm), 80 mg (533 ppm), and 100 mg (666 ppm) of catalyst were put into the reactor for photocatalytic degradation reaction. As shown in Figure 11a, the degradation performances after 120 min photocatalytic reaction were 55.7%, 100%, 98.8%, and 97.3%, respectively. Comparing the conditions of 30 mg (200 ppm) and 50 mg (333 ppm), it could be found that if more LaFe_0.7_Ni_0.3_O_3_ perovskite oxides were added to the solution, a better photocatalytic degradation performance would be obtained. However, while too many LaFe_0.7_Ni_0.3_O_3_ perovskite oxides were added, the excess catalyst would inhibit light penetration into the suspension. Thus, the availability of light energy was reduced, resulting in a reduction of the photocatalytic degradation capability [30]. Accordingly, the degradation performance was calculated by the 1st order kinetics, as shown in Figure 11b. It could be known that when the catalyst dosage was 30 mg (200 ppm), 50 mg (333 ppm), 80 mg (533 ppm), and 100 mg (666 ppm), their reaction rate constants *k* were 0.007, 0.0242, 0.0314, and 0.0506, respectively. Therefore, the pH of the solution was 3.5, and the catalyst dosage was 50 mg for the following examination.

**Figure 12:**
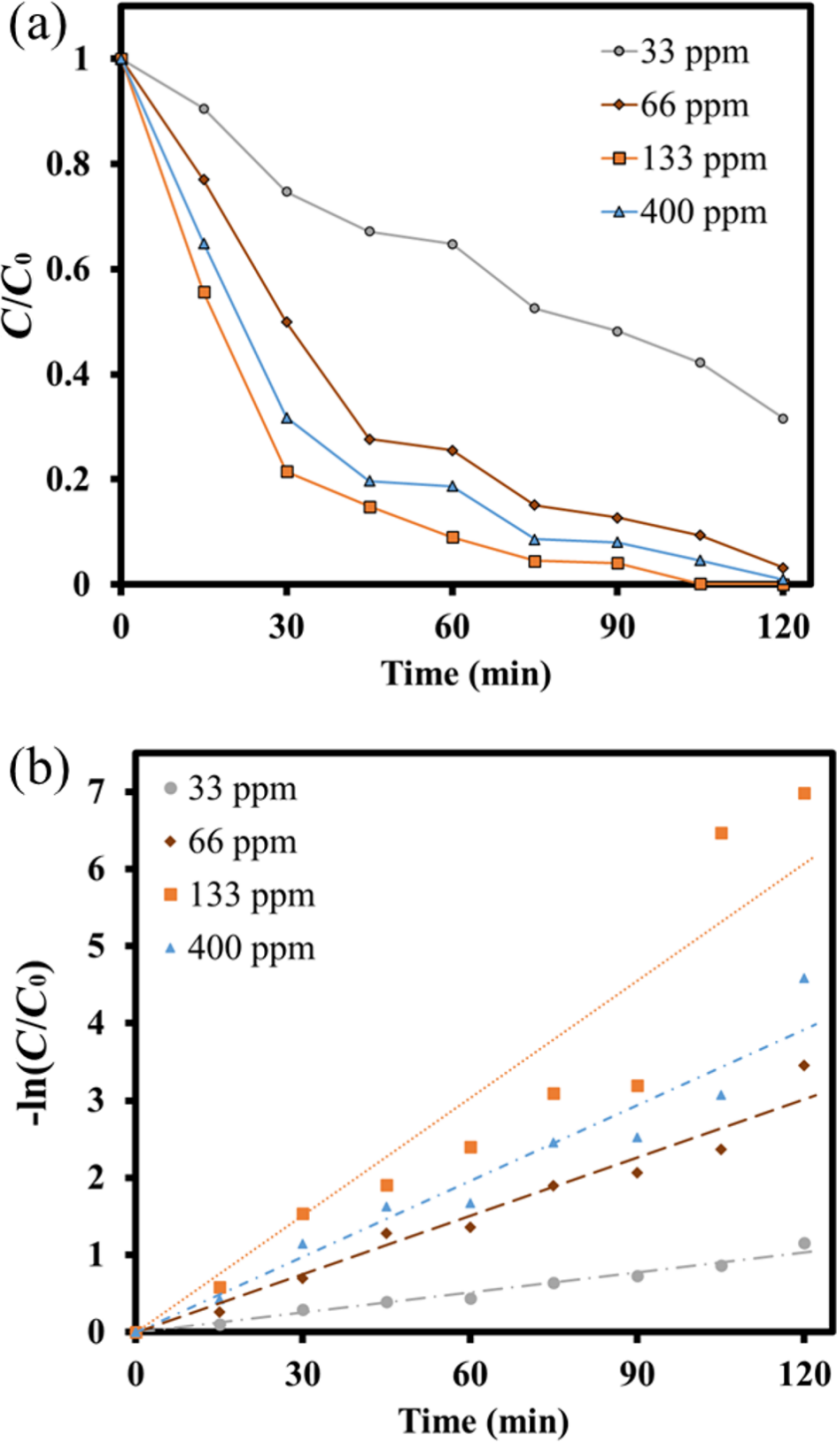
(a) The *C*/*C*_0_ and (b) 1st order kinetic analysis of the photodegradation using LaFe_0.7_Ni_0.3_O_3_ operating at different H_2_O_2_ addition.

Moreover, the amount of hydrogen peroxide added was manipulated in advance. Similarly, the amount of contaminant solution was 150 mL. The pH value of the solution was fixed at 3.5, the catalyst dosage was 50 mg, and hydrogen peroxide was added in various amounts, such as 5 µL (33 ppm), 10 µL (66 ppm), 20 µL (133 ppm) and 60 µL (400 ppm), respectively. The photocatalytic degradation experiment results were shown in Figure 12a. With the increase of hydrogen peroxide addition, the degradation capability after 120 min simulated solar light irradiation was 69.4%, 97.2%, 100%, and 99.9%, respectively. Then, their 1st order kinetics analysis was also depicted in Figure 12b. The reaction rate constants *k* of various conditions by adding different amounts of H_2_O_2_ were obtained as 0.0086, 0.0251, 0.0326, and 0.0506, respectively. It could be concluded that adding more H_2_O_2_ could increase the hydroxyl radicals in the aqueous solution and improve the performance of photocatalytic degrading pollutants. However, when too many hydroxyl radicals were generated, the hydroxyl radicals in the aqueous solution would combine with excess H_2_O_2_ to form superoxide hydrogen radicals and water [56], reducing the degradation capability.

Accordingly, the optimal operating conditions were summarized together: (1) the pH value of the aqueous solution was 3.5; (2) the amount of catalyst added was 50 mg in 150 mL solution (333 ppm); (3) the amount of hydrogen peroxide added was 20 µL. The MB removals contributed to the photocatalytic reactions and the Fenton reactions simutaneously. In order to further understand the effect of the photocatalysis and the Fenton reaction in the degradation reaction, some degradation tests were also carried out as controlling experiments for comparison, including: (1) without adding photocatalyst (No catalyst), (2) without light (No light), and (3) without adding H_2_O_2_ (No H_2_O_2_), as shown in Supporting Information File 1, Figure S4a. It could be observed that in the case of no catalyst added, the degradation was only 2.2% after 120 min, suggesting that H_2_O_2_ might not be easy to form ∙OH radicals under visible light irradiation to carry out the degradation reaction. Then, the degradation of the case without adding H_2_O_2_ was approximately 4.9% after 120 min. It indicated that the heterogeneous Fenton reaction might play a more critical role than the photocatalytic reaction. Another possibility, even though LaFe_0.7_Ni_0.3_O_3_ was a visible-light-driven photocatalyst, its electron–hole recombination was still too severe, resulting in relatively poor performance of MB removal. Next, the result of the case without light indicated the Fenton reaction was carried out without light. After 120 min, it was found that the degradation reached 69.2%. LaFe_0.7_Ni_0.3_O_3_ catalyst contained iron ions, which could certainly convert H_2_O_2_ into ∙OH and achieve a certain degree of degradation. Finally, compared with the above-mentioned controlling cases, the degradation under original conditions (All) reached 100% at 120 min, attributed mainly to the Fenton reaction and the photocatalysis, which could multiply the function of hydroxyl radicals. Their performances of MB degradation were calculated by 1st order kinetics, as shown in Figure S4b. The reaction rate constants *k* of different conditions: (1) No cat., (2) No H_2_O_2_, (3) No light, and (4) All, were 0.0002, 0.0005, 0.0096, and 0.0506, respectively. Moreover, the comparison of the prepared LaFe_0.7_Ni_0.3_O_3_ with other materials from the literature was also listed in Table S4. It showed that Fe_0.7_Ni_0.3_O_3_ samples revealed comparable photodegradation performance to other composite materials.

Moreover, the degradation of TC using LaFe_0.7_Ni_0.3_O_3_ prepared at pH 0 was also carried out under the optimal conditions of pH 3.5, catalyst = 333 ppm, and H_2_O_2_ = 20 µL as shown in Supporting Information File 1, Figure S5a. The TC concentration could drop by nearly 93.3% in 30 min of light irradiation compared with the initial concentration (20 ppm). The TC was degraded entirely in 60 min. Since there were multiple polar groups (hydroxyl group) within the chemical structure of TC [57], it was easy to attract the hydroxyl radicals in the TC solution, resulting in a better photocatalytic performance than MB. The kinetic rate constant of TC degradation was also calculated, as shown in Figure S5b. Accordingly, the reaction rate constants *k* of photodegrading TC and MB were 0.10991 and 0.0506, respectively, indicating LaFe_0.7_Ni_0.3_O_3_ revealed a great potential to decompose other organic pollutants.

## Conclusion

In this experiment, LaFe*_x_*Ni_1−_*_x_*O_3_ perovskite oxides were successfully synthesized by the sol–gel method. The calcination temperature and the pH value were manipulated during synthesizing LaFe*_x_*Ni_1−_*_x_*O_3_ photocatalysts. Through XRD, it could be found that the calcination temperature must be higher than 700 °C to reveal the clear crystal phase. Moreover, the LaFe*_x_*Ni_1−_*_x_*O_3_ photocatalysts prepared at pH 0 exhibited higher photocatalytic performance than at pH 7 due to more uniform structural features for the condition of pH 0. On the other hand, the Fe/Ni doping ratio during the synthesis was detected by XRD, indicating that the sample with the Fe/Ni ratio of 7/3 could obtain the largest crystal diameter with a better crystallinity. Meanwhile, the perovskite oxides with the Fe/Ni ratio of 7/3 had the highest specific surface area according to the BET BET measurement, which was conducive to photocatalysis. Furthermore, with changing the conditions during degradation, the pH value of the solution, the amount of catalyst dosage, and the amount of H_2_O_2_ added were examined. A higher pH value in the solution would cause hydrogen peroxide quickly decompose into oxygen and water. When the pH value was too low, the solution tended to produce too many hydrogen ions, which would combine with hydroxyl radicals to form water. An unsuitable pH value would decrease the photocatalytic degradation capability so that the operating pH was set at 3.5. Similarly, the catalyst could perform better with the increase of the additional amount. However, excess catalysts would inhibit light penetration and decrease light availability. Accordingly, the catalyst dosage was set at 50 mg. On the other hand, H_2_O_2_ could enhance the generation of the hydroxyl radicals in the aqueous solution. Unfortunately, when excess H_2_O_2_ produced too many hydroxyl radicals, the hydroxyl radicals would combine with H_2_O_2_ to form hydrogen peroxide radicals and water, reducing the poor photocatalytic degradation. The amount of H_2_O_2_ added was suggested at 20 µL. To sum up, the LaFe*_x_*Ni_1−_*_x_*O_3_ perovskite oxides could be the photocatalysts to conduct the photocatalytic Fenton reaction to decompose the MB pollutants under the irradiation of simulated AM 1.5G solar light. It could be seen that the LaFe*_x_*Ni_1−_*_x_*O_3_ perovskite oxides could be used as an environmentally to catalyze the photocatalytic degradation of pollutants.

## Experimental

### Materials

The precursors of LaFe*_x_*Ni_1−_*_x_*O_3_ for La, Fe, and Ni were lanthanum nitrate hexahydrate (99.9%, La(NO_3_)_3_·6H_2_O, Alfa Aesar), ferric nitrate nonahydrate (≥98.0%, Fe(NO_3_)_3_·9H_2_O, J.T. Baker), and nickel nitrate hexahydrate (98.0%, Ni(NO_3_)_2_·6H_2_O, Showa), respectively. The citric acid (95.0%, C_6_H_8_O_7_) and ammonia (28.0–30.0%, NH_4_OH) were both obtained from J.T. Baker Chemicals. Ethanol (95.0%, C_2_H_5_OH) was purchased from Echo Chemical. Hydrochloric acid (>37%, HCl) and hydrogen peroxide (50%, H_2_O_2_) were acquired from Honeywell Research Chemicals and OCI company, respectively. Model compounds, including methylene blue (≥98.0%, MB, C_16_H_18_N_3_SCl) and tetracycline (≥98.0%, TC, C_22_H_24_N_2_O_8_), were provided from Sigma-Aldrich. Commercial tungsten oxide (99.8%, WO_3_) was bought from Alfa Aesar.

### Synthesis of LaFe*_x_*Ni_1−_*_x_*O_3_

The LaFe*_x_*Ni_1−_*_x_*O_3_ catalysts were synthesized via the sol–gel method with citric acid crosslinking reaction, followed by self-propagating high-temperature synthesis. First, 0.02 mol lanthanum nitrate hexahydrate (La(NO_3_)_3_∙6H_2_O), ferric nitrate nonahydrate (Fe(NO_3_)_3_∙9H_2_O), and nickel nitrate hexahydrate (Ni(NO_3_)_2_∙6H_2_O) were dissolved in deionized water to form the mixed solution. Various photocatalysts with different molar ratios of Fe/Ni were manipulated at 0.1/0.9, 0.3/0.7, 0.5/0.5, 0.7/0.3, and 0.9/0.1. Accordingly, the chemical stoichiometric ratios of Fe and Ni were noted as *x* and 1−*x*, respectively. Second, 0.1 mol citric acid (C_6_H_8_O_7_) was also dissolved into the mixed solution as the cross-linking agent to gradually form the gel solution with light brown color. The pH value was adjusted to pH 0 or pH 7 with ammonia (NH_4_OH). The total volume of the brown solution was controlled at 100 mL, followed by being heated at 110 °C for 60 min with continuous stirring to remove water. Citric acid acted as a chelating agent during the dehydration process, so condensation and polymerization reactions occurred between citric acid and nitrate to chelate metal ions. Subsequently, the gel was formed and transferred to a high-temperature furnace for pre-calcination by self-propagating combustion in an air environment of 300 °C. The combustion duration was 20 min to remove the excess colloid of the gel and generate loose powders. After that, the obtained powder was ground with an agate grinder evenly and calcined under the air environment at different temperatures (500 °C, 600 °C, 700 °C, 800 °C) for 5 h. With further grinding uniformly, the LaFe*_x_*Ni_1−_*_x_*O_3_ perovskite oxides were prepared ultimately.

### Photocatalysts characterization

The LaFe*_x_*Ni_1−_*_x_*O_3_ photocatalysts were characterized by D8 advance (Bruker, Germany) to reveal the X-ray powder diffraction (XRD) patterns. The Cu Kα target was facilitated under 40 kV to generate the X-ray with λ = 0.15406 nm. The 2θ diffracting angle was set from 10° to 70° with a scan rate of 4 °/min. The crystalline sizes of the LaFe*_x_*Ni_1−_*_x_*O_3_ perovskite oxides were further obtained by Scherrer’s equation. To obtain the morphologies of the prepared samples, S-4800 (Hitachi, Japan) was employed to conduct field emission scanning electron microscopy (FESEM). The light absorption spectra of the perovskite oxides were inspected using V-670 (Jasco, Japan) to examine the UV–vis absorption capability with diffuse reflectance spectroscopy (DRS) from 200 to 800 nm. The nitrogen adsorption–desorption analyzer, ASAP 2020 PLUS (ASAP, USA), was applied to determine the specific surface area of the as-synthesized samples via the BET (Brunauer–Emmett–Teller) method. The calcination temperature of the perovskite oxides was determined via a Thermogravimetric Analyzer (SDT 2960, TA Instruments, USA).

### Photocatalytic MB and tetracycline degradation

The photocatalytic activity of LaFe*_x_*Ni_1−_*_x_*O_3_ photocatalysts was examined by evaluating the performance of photocatalytic MB degradation. 300 W Xe-lamp equipped with AM 1.5G filter (Newport Corporation, USA) was used as the light source in the degradation experiment. The light intensity was adjusted to 100 mW/cm^2^ calibrated with a light intensity meter Model 1919-R (Newport Corporation, USA). First, 150 mL of 20 ppm MB solution was prepared and transferred to a quartz reactor. Perovskite oxide photocatalysts with 30 mg, 50 mg, 80 mg, and 100 mg were placed into the reactor. Furthermore, different amounts of 50% H_2_O_2_ (5 µL, 10 µL, 20 µL, and 60 µL) were also transferred into the reactor via micropipettes. The pH of the reactor solution was adjusted to 1.5, 3.5, and 5.5 by adding hydrochloric acid. Then, the photocatalysts were deposited into the reactor with the magnet stirring at 600 rpm. Before light irradiation, the MB concentration reached adsorption–desorption equilibrium in 30 min in the dark, which was recorded (*C*_0_). After that, the simulated AM 1.5G solar light was turned on, and the photocatalytic reaction occurred. The MB concentrations (C) were monitored every 15 min by sampling 5 mL of solution, then filtering the samples through a 0.22 µm needle filter. The MB concentrations were evaluated using the UV–vis spectrometer U-2910 (HITACHI, Japan). The detection range was set from 200 to 800 nm. The scanning rate was 400 nm/min. The maximum absorption peaks of MB and TC solution were at 664 nm and 356 nm, respectively.

## Supporting Information

Figure S1: The nitrogen adsorption and desorption curves of the samples with different Fe/Ni synthesis ratios at pH 0. Figure S2: Pore size distribution of the samples with different Fe/Ni synthesis ratios. Figure S3: The FESEM images of LaFe*_x_*Ni_1−_*_x_*O_3_ prepared at pH 7 (at the magnification of 100,000×). Figure S4: (a) The *C*/*C*_0_ and (b) the 1st order kinetic analysis of the photodegradation using LaFe_0.7_Ni_0.3_O_3_ operating at different controlling conditions. Figure S5: (a) The *C*/*C*_0_ and (b) 1st order kinetic analysis of the MB and TC photodegradation using LaFe_0.7_Ni_0.3_O_3_ operating at the optimal condition. Table S1: The specific surface area, pore size, and pore volume for the samples with different Fe/Ni ratios. Table S2: The unit cell parameters and cell volume for the samples with different Fe/Ni ratios. Table S3: The detailed EDS information of various samples with different Fe/Ni ratios. Table S4: The comparison of photodegradation performance over various LaFeO_3_-related samples.

File 1 Additional figures and tables.

## References

[1] Otto B, Schleifer L (2020). Domestic Water Use Grew 600% Over the Past 50 Years.

[2] Searchinger T, Waite R, Hanson C (2018). Creating a Sustainable Food Future.

[3] Lin Y-T, Wang Y-H, Wu J C S, Wang X (2021). Appl Catal, B.

[4] Lin Y-T, Huang C-W, Wang Y-H, Wu J C S (2020). Top Catal.

[5] Gatidou G, Kinyua J, van Nuijs A L N, Gracia-Lor E, Castiglioni S, Covaci A, Stasinakis A S (2016). Sci Total Environ.

[6] Rott U, Minke R (1999). Water Sci Technol.

[7] Choudhary S, Silakari O (2018). Thiazine: A Versatile Heterocyclic Scaffold for Multifactorial Diseases. Key Heterocycle Cores for Designing Multitargeting Molecules.

[8] Zhang Q, Jiang L, Wang J, Zhu Y, Pu Y, Dai W (2020). Appl Catal, B.

[9] Shao S, Hu Y, Cheng J, Chen Y (2018). Crit Rev Biotechnol.

[10] Jung C, Son A, Her N, Zoh K-D, Cho J, Yoon Y (2015). J Ind Eng Chem (Amsterdam, Neth).

[11] Chu K H, Al-Hamadani Y A J, Park C M, Lee G, Jang M, Jang A, Her N, Son A, Yoon Y (2017). Chem Eng J.

[12] Monteil H, Pechaud Y, Oturan N, Trellu C, Oturan M A (2021). Chem Eng J.

[13] Kumar A, Rana A, Sharma G, Naushad M, Dhiman P, Kumari A, Stadler F J (2019). J Mol Liq.

[14] Souza F S, da Silva V V, Rosin C K, Hainzenreder L, Arenzon A, Féris L A (2018). Environ Technol.

[15] Huang C-W, Sin W-C, Nguyen V-H, Wu Y-C, Chen W-Y, Chien A C (2020). Top Catal.

[16] Deng Y (2009). Int J Environ Waste Manage.

[17] Hakika D C, Sarto S, Mindaryani A, Hidayat M (2019). Catalysts.

[18] Fenton H J H (1894). J Chem Soc, Trans.

[19] Bauer R, Waldner G, Fallmann H, Hager S, Klare M, Krutzler T, Malato S, Maletzky P (1999). Catal Today.

[20] Zhang M-h, Dong H, Zhao L, Wang D-x, Meng D (2019). Sci Total Environ.

[21] Do H-T, Phan Thi L-A, Dao Nguyen N H, Huang C-W, Le Q V, Nguyen V-H (2020). J Chem Technol Biotechnol.

[22] Huang C-W, Wu M-C (2020). J Chem Technol Biotechnol.

[23] Li L, Wang X, Zhang Y (2014). Mater Res Bull.

[24] Ye Y, Yang H, Wang X, Feng W (2018). Mater Sci Semicond Process.

[25] Wang K, Niu H, Chen J, Song J, Mao C, Zhang S, Gao Y (2017). Appl Surf Sci.

[26] Guan S, Yang H, Sun X, Xian T (2020). Opt Mater (Amsterdam, Neth).

[27] Ye Y, Yang H, Zhang H, Jiang J (2020). Environ Technol.

[28] Orak C, Atalay S, Ersöz G (2017). Sep Sci Technol.

[29] Garcia-Muñoz P, Fresno F, Lefevre C, Robert D, Keller N (2020). Catal Sci Technol.

[30] Jauhar S, Dhiman M, Bansal S, Singhal S (2015). J Sol-Gel Sci Technol.

[31] Phan T T N, Nikoloski A N, Bahri P A, Li D (2018). J Ind Eng Chem (Amsterdam, Neth).

[32] Pecchi G, Reyes P, Zamora R, Cadús L E, Fierro J L G (2008). J Solid State Chem.

[33] Gallego J, Mondragon F, Batiot-Dupeyrat C (2013). Appl Catal, A.

[34] Zheng D, Wei G, Xu L, Guo Q, Hu J, Sha N, Zhao Z (2019). J Photochem Photobiol, A.

[35] Provendier H, Petit C, Estournes C, Kiennemann A (1998). Stud Surf Sci Catal.

[36] Provendier H, Petit C, Kiennemann A (2001). C R Acad Sci, Ser IIc: Chim.

[37] Jahangiri A, Aghabozorg H, Pahlavanzadeh H (2013). Int J Hydrogen Energy.

[38] Ghorai K, Panda A, Hossain A, Bhattacharjee M, Chakraborty M, Bhattacharya S K, Show B, Sarkar A, Bera P, Kim H (2022). J Rare Earths.

[39] Iqbal S, Bibi I, Majid F, Kamal S, Alwadai N, Iqbal M (2022). Opt Mater (Amsterdam, Neth).

[40] ZHANG L-f, WANG Y-p, HUANG Q-w (2009). Trans Nonferrous Met Soc China.

[41] Li Y, Yao S, Wen W, Xue L, Yan Y (2010). J Alloys Compd.

[42] Monshi A, Foroughi M R, Monshi M R (2012). World J Nano Sci Eng.

[43] Gong S, Xie Z, Li W, Wu X, Han N, Chen Y (2019). Appl Catal, B.

[44] Wiranwetchayan O, Promnopas S, Phadungdhitidhada S, Phuruangrat A, Thongtem T, Singjai P, Thongtem S (2019). Ceram Int.

[45] Khan A, Hussain R, Toufiq A M, Shah A, Khan B A, Niaz Z, Rahman S u (2020). Mater Charact.

[46] Chen C, Zhou J, Geng J, Bao R, Wang Z, Xia J, Li H (2020). Appl Surf Sci.

[47] Shi J, Chang Y, Tang Y, Wang X, Wang X, Zhang X, Cao J (2020). Ceram Int.

[48] Mehdizadeh P, Amiri O, Rashki S, Salavati-Niasari M, Salimian M, Foong L K (2020). Ultrason Sonochem.

[49] Hoseini A-A, Farhadi S, Zabardasti A, Siadatnasab F (2019). RSC Adv.

[50] Pan G-T, Chong S, Pan K-L, Chang M-B, Yang T C-K, Shukla P (2017). Clean Technol Environ Policy.

[51] Walling C (1975). Acc Chem Res.

[52] Yao Y, Chen H, Lian C, Wei F, Zhang D, Wu G, Chen B, Wang S (2016). J Hazard Mater.

[53] Strlič M, Kolar J, Šelih V-S, Kočar D, Pihlar B (2003). Acta Chim Slov.

[54] Taran O P, Ayusheev A B, Ogorodnikova O L, Prosvirin I P, Isupova L A, Parmon V N (2016). Appl Catal, B.

[55] Wang S (2008). Dyes Pigm.

[56] Zong X, Wu G, Yan H, Ma G, Shi J, Wen F, Wang L, Li C (2010). J Phys Chem C.

[57] Yu X, He J, Zhang Y, Hu J, Chen F, Wang Y, He G, Liu J, He Q (2019). J Alloys Compd.

