# Supplementary material for: Solar-light-driven LaFexNi1−xO3 perovskite oxides for photocatalytic Fenton-like reaction to degrade organic pollutants
Source: Beilstein J Nanotechnol. 2022 Sep 5;13:882–95. doi: 10.3762/bjnano.13.79 (PMC9475182; doi:10.3762/bjnano.13.79)
Supplement: File 1 — Additional figures and tables. [file Beilstein_J_Nanotechnol-13-882-s001.pdf]

BEILSTEIN JOURNAL OF NANOTECHNOLOGY

Supporting Information

for

Solar-light-driven $LaFe_xNi_{1-x}O_3$ perovskite oxides for photocatalytic Fenton-like reaction to degrade organic pollutants

Chao-Wei Huang, Shu-Yu Hsu, Jun-Han Lin, Yun Jhou, Wei-Yu Chen, Kun-Yi Andrew Lin, Yu-Tang Lin and Van-Huy Nguyen

Beilstein J. Nanotechnol. **2022,** *13,* 882–895. doi:10.3762/bjnano.13.79

Additional figures and tables

License and Terms: This is a supporting information file under the terms of the Creative Commons Attribution License (https://creativecommons.org/licenses/by/4.0). Please note that the reuse, redistribution and reproduction in particular requires that the author(s) and source are credited and that individual graphics may be subject to special legal provisions.
The license is subject to the *Beilstein Journal of Nanotechnology* terms and conditions: (https://www.beilstein-journals.org/bjnano/terms)

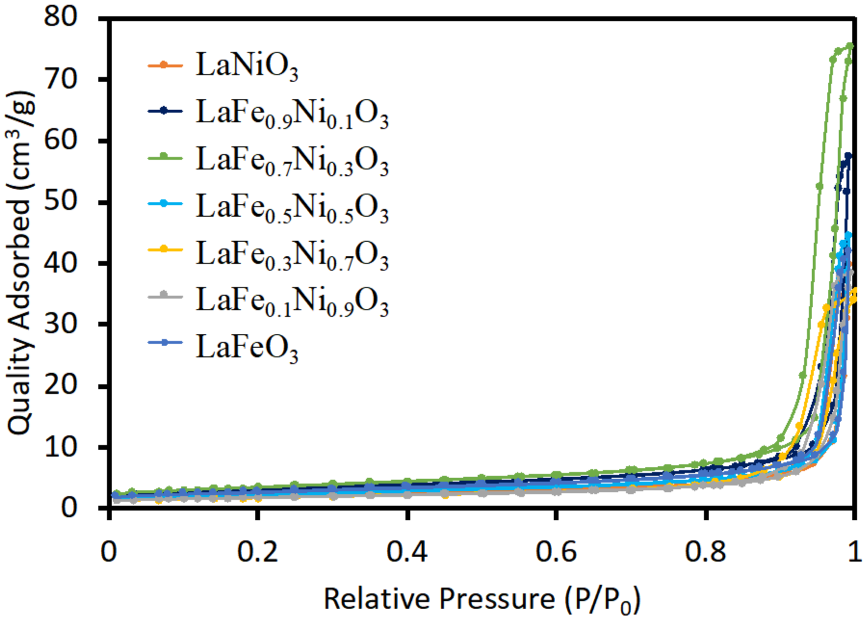

Figure S1: The nitrogen adsorption and desorption curves of the samples with different Fe/Ni synthesis ratios at pH 0.

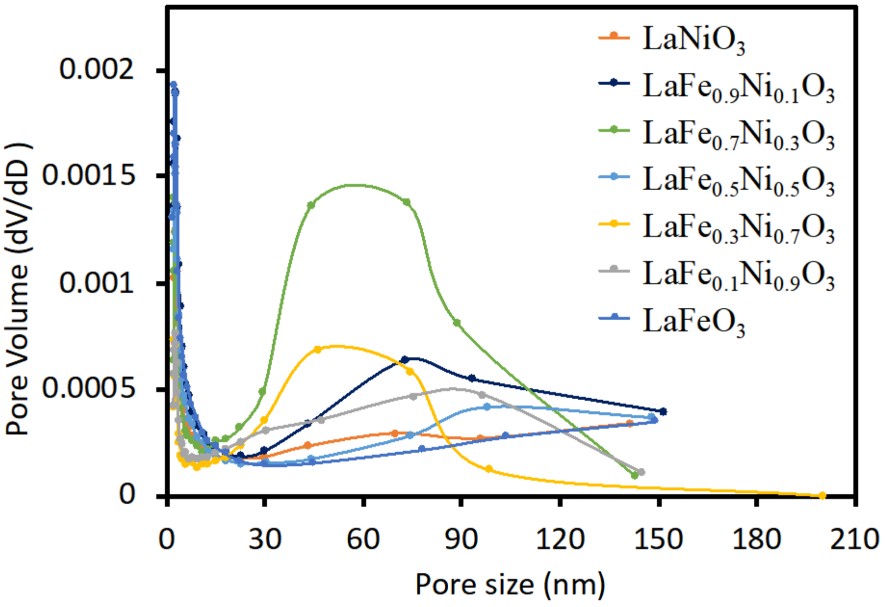

Figure S2: Pore size distribution of the samples with different Fe/Ni synthesis ratios.

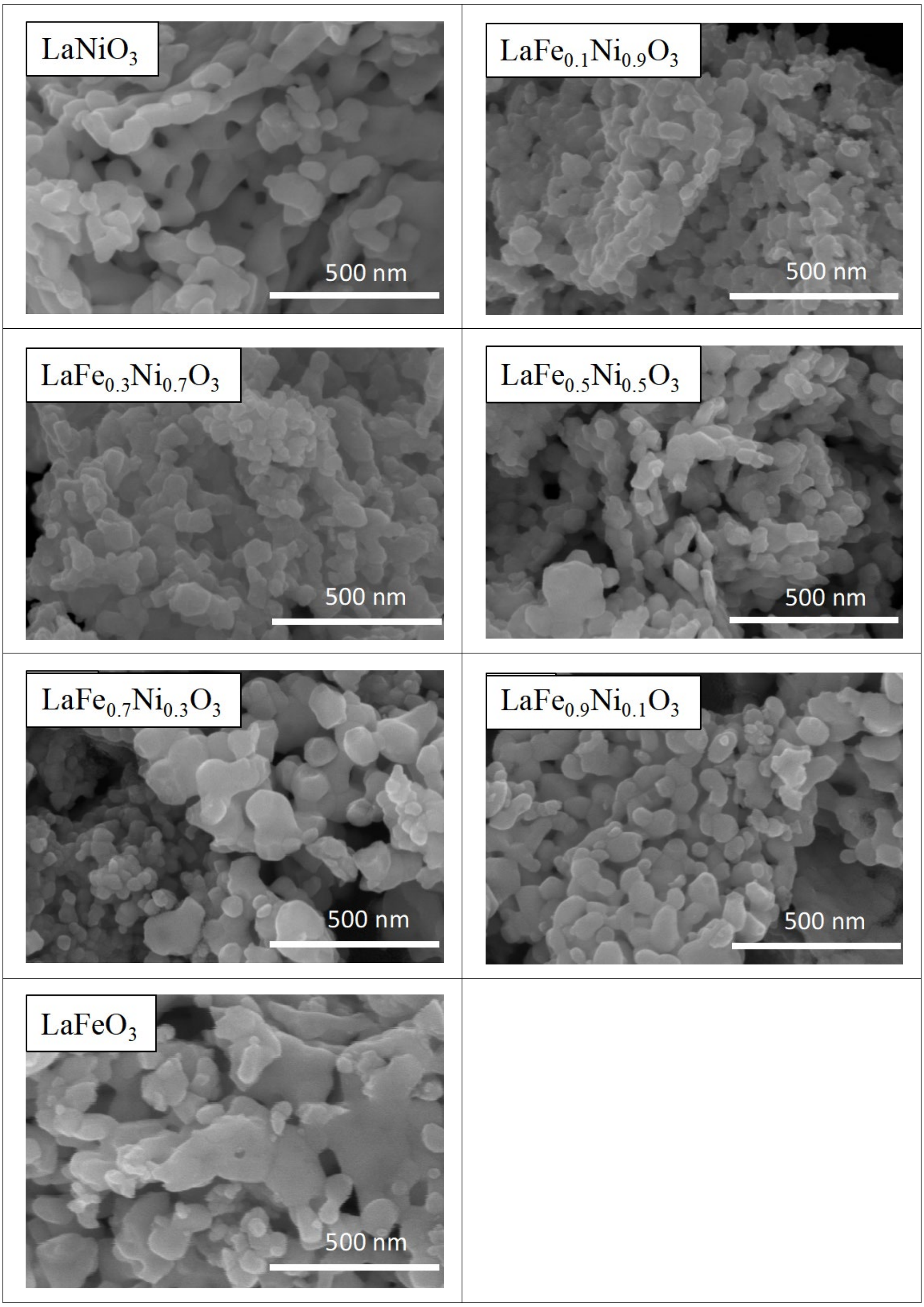

Figure S3: FESEM images of $LaFe_xNi_{1-x}O_3$ prepared at pH 7 (at the magnification of 100,000×).

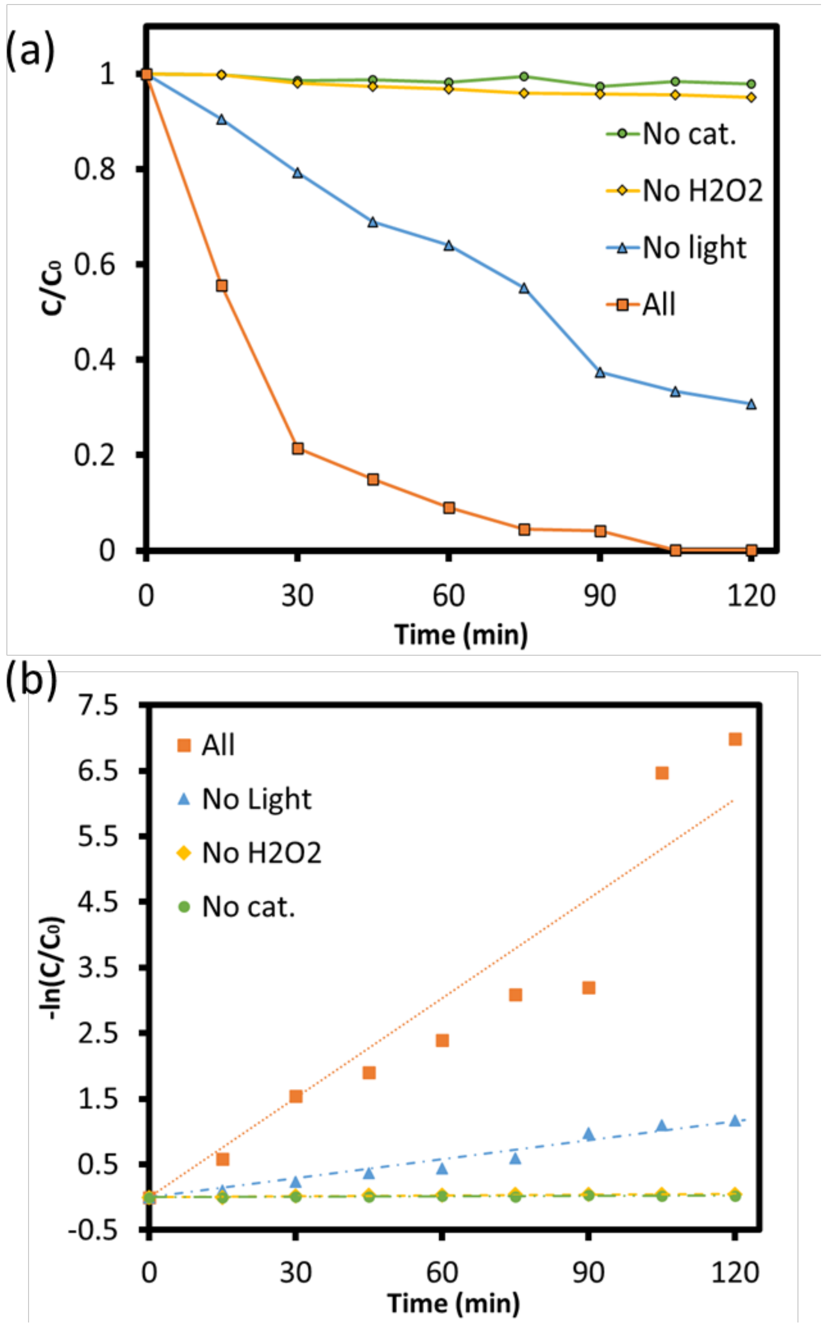

Figure S4: (a) C/C_0 and (b) the 1st order kinetic analysis of the photodegradation using $LaFe_{0.7}Ni_{0.3}O_3$ operating at different controlling conditions.

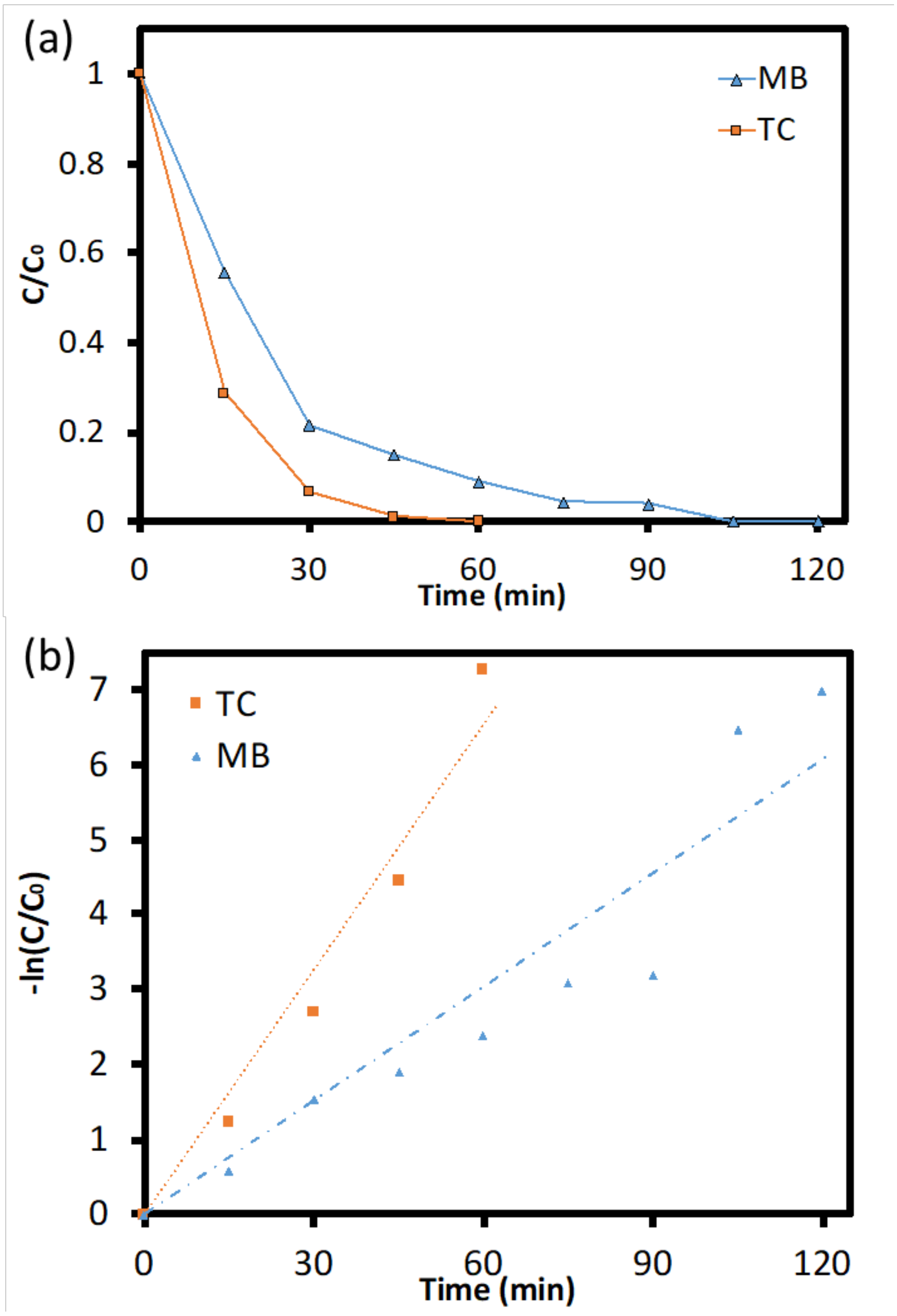

Figure S5: (a) C/C_0 and (b) 1st order kinetic analysis of the MB and TC photodegradation using $LaFe_{0.7}Ni_{0.3}O_3$ operating at the optimal condition.

Table S1: The specific surface area, pore size, and pore volume for the samples with different Fe/Ni ratios.

**Samples with Fe/Ni ratios**	**0/1**	**1/9**	**3/7**	**5/5**	**7/3**	**9/1**	**1/0**
Surface Area (m^2/g)	7.18	6.25	6.17	8.35	12.30	10.79	9.82
Pore Size (nm)	26.73	35.72	34.05	28.49	36.71	29.62	18.33
Pore Volume (cm^3/g)	0.0480	0.0558	0.0525	0.0595	0.1129	0.0799	0.0450

Table S2: The unit cell parameters and cell volume for the samples with different Fe/Ni ratios.

**Samples**	**Cell parameter (Å)**			**Cell volume (Å^3)**
	**a**	**b**	**c**	
$LaFeO_3$	5.592	7.854	5.044	221.530
$LaFe_{0.9}Ni_{0.1}O_3$	5.579	7.828	5.53	241.508
$LaFe_{0.7}Ni_{0.3}O_3$	5.601	7.844	5.545	243.615
$LaFe_{0.5}Ni_{0.5}O_3$	5.537	7.776	5.48	235.945
$LaFe_{0.5}Ni_{0.5}O_3$	5.502		6.762	177.269
$LaFe_{0.3}Ni_{0.7}O_3$	5.511		6.712	176.534
$LaFe_{0.1}Ni_{0.9}O_3$	5.491		6.443	168.232
$LaNiO_3$	5.458		6.614	170.627

Table S3: The detailed EDS information of various samples with different Fe/Ni ratios.

Sample	Element symbol	Element name	Atomic %	Weight %	Detected Fe/Ni	Designed Fe/Ni
$LaNiO_3$	O	Oxygen	40.07	12.62	0	0
	La	Lanthanum	21.63	59.17		
	Ni	Nickel	20.83	24.07		
	C	Carbon	17.48	4.13		
$LaFe_{0.1}Ni_{0.9}O_3$	C	Carbon	40.97	12.91	0.11	0.11
	O	Oxygen	32.54	13.66		
	La	Lanthanum	15.56	56.68		
	Ni	Nickel	9.83	15.13		
	Fe	Iron	1.11	1.62		
$LaFe_{0.3}Ni_{0.7}O_3$	C	Carbon	38.28	11.65	0.41	0.43
	O	Oxygen	34.04	13.8		
	La	Lanthanum	16.53	58.2		
	Ni	Nickel	7.88	11.73		
	Fe	Iron	3.26	4.62		
$LaFe_{0.5}Ni_{0.5}O_3$	O	Oxygen	38.44	11.67	0.97	1.00
	La	Lanthanum	24.73	65.19		
	C	Carbon	19.66	4.48		
	Ni	Nickel	8.7	9.69		
	Fe	Iron	8.46	8.97		
$LaFe_{0.7}Ni_{0.3}O_3$	O	Oxygen	42.08	12.51	2.31	2.33
	La	Lanthanum	25.49	65.78		

	C	Carbon	15.01	3.35		
	Fe	Iron	12.15	12.61		
	Ni	Nickel	5.27	5.75		
$LaFe_{0.9}Ni_{0.1}O_3$	O	Oxygen	53.02	22.03	8.75	9.00
	C	Carbon	20.51	6.4		
	La	Lanthanum	15.35	55.37		
	Fe	Iron	9.98	14.47		
	Ni	Nickel	1.14	1.74		
$LaFeO_3$	O	Oxygen	37.46	15.75	-	-
	C	Carbon	36.02	11.37		
	La	Lanthanum	15.57	56.82		
	Fe	Iron	10.95	16.06		

Table S4: The comparison of photodegradation performance over various $LaFeO_3$-related samples.

Catalyst	H_2O_2 concentration	Light source	MB concentration	performance	Ref.
Cds/$LaFeO_3$	4.0 mM	400 W Hg lamp	25 mg/L	180 min 95%	[1]
$LaFeO_3$/TiO_2 NTAs	N/A	500 W tungsten halogen lamp	10 mg/L	120 min 70%	[2]
AgCl/Ag/$LaFeO_3$	N/A	400 W Xenon lamp	20 mg/L	100 min 98%	[3]
$LaFeO_3$/g-C_3N_4	N/A	3 W LED	5 mg/L	120 min 95%	[4]
$LaFe_{0.7}Ni_{0.3}O_3$	4.0 mM	300 W Xenon lamp	10 mg/L	120 min 100%	This work

References

1. Shao, S.; Hu, Y.; Cheng, J.; Chen, Y. *Crit. Rev. Biotechnol.* **2018,** *38,* 1195–1208. doi:10.1080/07388551.2018.1471038
2. Jung, C.; Son, A.; Her, N.; Zoh, K.-D.; Cho, J.; Yoon, Y. *J. Ind. Eng. Chem. (Amsterdam, Neth.)* **2015,** *27,* 1–11. doi:10.1016/j.jiec.2014.12.035
3. Chu, K. H.; Al-Hamadani, Y. A. J.; Park, C. M.; Lee, G.; Jang, M.; Jang, A.; Her, N.; Son, A.; Yoon, Y. *Chem. Eng. J.* **2017,** *327,* 629–647. doi:10.1016/j.cej.2017.06.137

4. Monteil, H.; Pechaud, Y.; Oturan, N.; Trellu, C.; Oturan, M. A. *Chem. Eng. J.* **2021,** *404,* 127048. doi:10.1016/j.cej.2020.127048
